# Laboratory colonization and mass rearing of phlebotomine sand flies (Diptera, Psychodidae)

**DOI:** 10.1051/parasite/2017041

**Published:** 2017-11-15

**Authors:** Phillip Lawyer, Mireille Killick-Kendrick, Tobin Rowland, Edgar Rowton, Petr Volf

**Affiliations:** 1 Monte L. Bean Life Science Museum, Brigham Young University, 2103 MLBM, Provo, UT 84602 USA; 2 2 Place du Temple, 30440 Sumène France; 3 Division of Entomology, Walter Reed Army Institute of Research, 503 Robert Grant Ave., Silver Spring, MD 84910 USA; 4 Department of Parasitology, Faculty of Sciences, Charles University in Prague, Vinicna 7, 128 44, Praha Czech Republic

**Keywords:** *Phlebotomus*, *Lutzomyia*, sand fly colony, leishmaniasis, mass rearing

## Abstract

Laboratory colonies of phlebotomine sand flies are necessary for experimental study of their biology, behaviour and mutual relations with disease agents and for testing new methods of vector control. They are indispensable in genetic studies and controlled observations on the physiology and behaviour of sand flies, neglected subjects of high priority. Colonies are of particular value for screening insecticides. Colonized sand flies are used as live vector models in a diverse array of research projects, including xenodiagnosis, that are directed toward control of leishmaniasis and other sand fly-associated diseases. Historically, labour-intensive maintenance and low productivity have limited their usefulness for research, especially for species that do not adapt well to laboratory conditions. However, with growing interest in leishmaniasis research, rearing techniques have been developed and refined, and sand fly colonies have become more common, enabling many significant breakthroughs. Today, there are at least 90 colonies representing 21 distinct phlebotomine sand fly species in 35 laboratories in 18 countries worldwide. The materials and methods used by various sand fly workers differ, dictated by the availability of resources, cost or manpower constraints rather than choice. This paper is not intended as a comprehensive review but rather a discussion of methods and techniques most commonly used by researchers to initiate, establish and maintain sand fly colonies, with emphasis on the methods proven to be most effective for the species the authors have colonized. Topics discussed include collecting sand flies for colony stock, colony initiation, maintenance and mass-rearing procedures, and control of sand fly pathogens in colonies.

## Abstract (fr)

Les colonies de laboratoire de phlébotomes sont nécessaires pour une étude expérimentale de leur biologie, leur comportement et leurs relations mutuelles avec des agents pathogènes et pour tester de nouvelles méthodes de lutte antivectorielle. Elles sont indispensables dans les études génétiques et les observations contrôlées sur la physiologie et le comportement des phlébotomes, sujets négligés de haute priorité. Les colonies ont une valeur particulière pour le criblage des insecticides. Les phlébotomes en élevage sont utilisés comme modèles de vecteurs vivants dans un éventail varié de projets de recherche, y compris le xénodiagnostic, qui visent le contrôle de la leishmaniose et d'autres maladies associées aux phlébotomes. Historiquement, la maintenance à forte intensité de main-d'œuvre et la faible productivité ont limité leur utilité pour la recherche, en particulier pour les espèces qui ne s'adaptent pas bien aux conditions de laboratoire. Mais, avec un intérêt croissant pour la recherche sur la leishmaniose, les techniques d'élevage ont été développées et affinées, et les colonies de phlébotomes sont devenues plus fréquentes, permettant de nombreuses percées significatives. Aujourd'hui, il y a au moins 90 colonies représentant 21 espèces distinctes de phlébotomes dans 35 laboratoires et 18 pays à travers le monde. Les matériaux et les méthodes utilisés par divers chercheurs sur les phlébotomes diffèrent, dictés par la disponibilité des ressources et les contraintes de coûts ou de main-d'œuvre plutôt que par le choix. Cet article n'est pas destiné à être un examen complet, mais plutôt une discussion sur les méthodes et les techniques les plus utilisées par les chercheurs pour initier, établir et maintenir les colonies de phlébotomes, en mettant l'accent sur les méthodes démontrées les plus efficaces pour les espèces que les auteurs ont établies en colonies. Les sujets abordés comprennent la collecte de phlébotomes pour les stocks de colonies, l'initiation des colonies, les procédures de maintenance et d'élevage et le contrôle des agents pathogènes des phlébotomes dans les colonies.

## Dedication

While many have made significant contributions to the study of phlebotomine sand flies and their colonization in the laboratory, the authors wish to remember in particular Professor Robert Killick-Kendrick and Professor Jean-Antoine Rioux. Professor Killick-Kendrick was the great protagonist of the establishment of laboratory phlebotomine sand fly colonies, which led to fundamental studies of *Leishmania*-vector interactions. The contributions of Jean-Antoine Rioux to this area of research are likewise remarkable for numerous studies performed in the mid-sixties in the Cévennes region of France and the Maghreb of North Africa, at a time when leishmaniasis was still a rather neglected disease and very little work had been done on the vectors. Not only were these close accomplices exceptional scientists but, more importantly, they were superb mentors and cherished friends.

## Table of contents

Introduction
Collecting wild-caught sand flies for colony stock
  Species of interest
  Selecting the collection site
  Collecting methods
  Processing collected flies in the field
Initiating the laboratory colony
  Overview of sand fly biology
  Isoline Rearing and Species Identification
  Life-Table Studies
Colony Maintenance Procedures
  Rearing Conditions
  Adult Holding and Mating Cages
  Adults
  Rearing immature stages
  Adult emergence
Parasites and pathogens
  Gregarines
  Microsporidians
  Mites
  Fungal and bacterial infections
  Nematodes
Good Housekeeping and Cleanlines
Disclaimers
References

## Introduction

**Figure 1:**
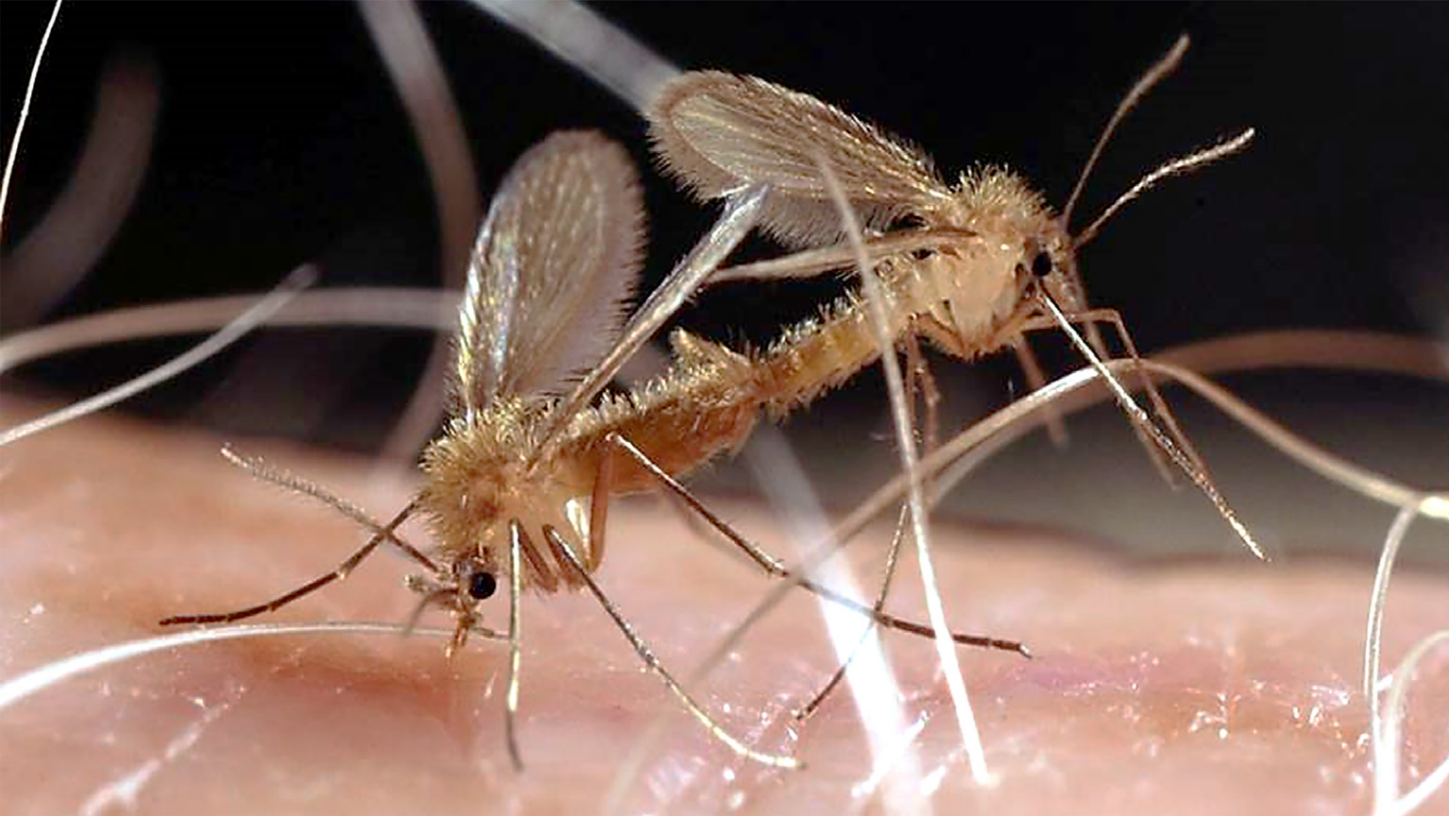
*Phlebotomus duboscqi* Neveu-Lemaire mating pair (Photo by E. Rowton). Colonized flies of this and other species are used as live vector models in research directed toward control of leishmaniasis and other sand fly-associated diseases.

The importance of establishing and maintaining large laboratory colonies of phlebotomine sand flies was summarized by Safyanova [56] as “necessary for the experimental study of their biology, behaviour and mutual relations with disease agents, and for the testing of new methods of vector control.” The WHO Scientific Working Group on leishmaniasis added the following emphasis: “Colonies are valuable in work on vector potential, life cycles of *Leishmania* and transmission by bite. They are indispensable in genetic studies and in controlled observations on the physiology and behaviour of sand flies, all of which are neglected subjects of high priority. Colonies are of particular value for screening insecticides.” [4] Colonized sand flies are used as live vector models in a diverse array of research projects directed toward control of leishmaniasis and other sand fly-associated diseases (Figure 1). They are essential in xenodiagnosis studies to establish whether human subjects with post-kala-azar dermal leishmaniasis (PKDL) and asymptomatic infections serve as reservoirs of *Leishmania donovani* (Laveran & Mesnil, 1903) infection, contributing to disease transmission and potentially influencing development of public health policy. However, until the early 1980s, fewer than a dozen closed colonies of about six species of sand flies were available to researchers for experimental use [11,18–20,22,31,55,63]. The pioneering studies of Killick-Kendrick, Laney and Ready on the establishment, maintenance and productivity of a laboratory colony of *Lutzomyia longipalpis* Lutz and Neiva 1912 [31] provided significant impetus for others to establish colonies of several sand fly species from both the Old and New World. Nonetheless, complex and labour-intensive maintenance procedures and low productivity limited their usefulness for leishmaniasis research, and still do, especially for species that do not adapt well to laboratory conditions. Although at least 50 species of sand flies have been colonized using various techniques, many were only temporary efforts and less than half have been mass-reared successfully as closed colonies for research purposes [5,6,11,12,15,16,18–20,25,28,31,38,39,44,50,51,57,59,63]. With growing interest in leishmaniasis research, rearing techniques have been developed and refined, and laboratory sand fly colonies have become more common, robust and useful, enabling many significant breakthroughs [24,26,27,33,38,42,46,48,49,59]. Currently, there are 90 colonies representing 21 distinct species of phlebotomine sand flies in 35 laboratories located in 18 countries worldwide that are registered in the Global Sand Fly Colony Database (Table 1). Readers who maintain sand fly colonies that are not included in this database are encouraged to register their colonies by contacting Dr Phillip Lawyer at (plawyer349@verizon.net). Materials and methods used by various sand fly workers differ between laboratories, often dictated by availability of resources, cost, or manpower constraints rather than choice. This supplement is not intended to be a comprehensive review but rather a discussion of methods and techniques most effectively used by researchers to initiate, establish and maintain sand fly colonies, with emphasis on those proven to be most effective for species the authors have colonized. Nonetheless, it should be noted that not all species respond equally well to these methods and specific modifications may be necessary to accommodate the peculiarities of a particular sand fly species. Also reported herein are results of experiments conducted in our laboratories and elsewhere that have led to significant improvements in mass-rearing efficiency and productivity.

**Table 1:** Global sand fly colony database-abbreviated (* indicates unconfirmed/not updated).

Country	Laboratory and Postal Address	Investigator(s)	Email Addresses	Species	Site of Origin
Bangladesh	Parasitology Unit, International Centre for Diarrhoeal Disease Research, Mohakhali, Dhaka-12, Bangladesh	Debashis Ghosh	deba.du@yahoo.com	*Phlebotomus argentipes*	Bangladesh

Brazil	Laboratório de ecologia de Doenças Transmissíveis na Amazônia, Res. Prog. Infect. Dis. Ecology in the Amazon	Felipe Pessoa	felipe.pessoa@fiocruz.br facoessia@gmail.com	*Lutzomyia migonei* *Lutzomyia longipalpis*	Baturité, Cerará, Brzail Santerém, Pará, Brazil
	Instituto Leonidas e Maria Deane, Fiocruz Rua Teresina, 476. Adrianopolis, Manaus-AM. CEP: 69.057-070, Brazil			* *	
					
	Laboratorio Inderdisciplinar em Vigilancia Entomologia: Diptera e Hemiptera,	Elizabeth F. Rangel, Nataly A. Souza,	efrangel@gene.dbbm.fiocruz.br souzana@ioc.fiocruz.br	*Lutzomyia longipalpis* *Lutzomyia longipalpis* *Lutzomyia longipalpis*	Abaetetuba, Brazil Cometa, Brazil Barcarena, Brazil
	Instituto Oswaldo Cruz, Fiocruz,			* *	
	Av. Brasil n^o^ 4365 Pav. Carlos Chagas, CDP 21045 − 900, Rio de Janeiro, RJ, Brazil				

	Laboratory of Medical Entomology,	Paulo F. P. Pimenta	pimenta@cpqrr.fiocruz.br	*Lutzomyia longipalpis*	Lapinha Cave, MG, Brazil
	Centro de Pesquisas Ren Rachou − Fiocruz	Edelberto Santos-Dias	edel@cpqrr.fiocruz.br		
	Av. Augusto de Lima, 1715 − Barro Preto, CEP 30091-493 Belo Horizonte, MG, Brazil	Fabiana de Olivera Lara de Silva	fabiana@cpqrr.br		
					
	Laboratorio de Leishmanioses, Hospital de Doencas Infecto-Contagiosas	Carlos H. Nery Costa	costa@ranet.com.br	*Lutzomyia longipalpis*	Teresina, PI, Brazil
	Rua Gov. Artu de Vasconcelos 151-Sul				
	64000-450 Teresina, PI, Brazil				

	Laboratorio de Doencas Parasitarias	Reginaldo P. Brazil	brazil.reginaldo@gmail.com	*Lutzomyia longipalpis*	Serra da Tiririca,
	Instituto Oswaldo Cruz-Fiocruz,				Niteroi, RJ, Brazil
	Av. Brasil 4365 − Manguinhos, Rio −21045-900, Brazil				

	Universidade Estadual Paulista Julio de Mewquita Filho, Araraguara, Faculdade de Ciencias Biologicas, Departmento de Ciencas, Biologicas, Rodovia Araraguara Jau, Km 01 −s/n, Campos Ville − Araraguara, SPCEP: 14.800-903, Brazil	Mara Christina Pinto Thais Marchi Goulart Flavia Benini da Rocha Silva	marap@fcfar.unesp.br thamarchi@gmail.com flavinhabenini@gmail.com	*Nyssomyia neivai*	Sao Carlos, Sao Paulo, Brazil

	Laboratorio de Biologia Molecular de Parasitos e Vetores, Instituto Oswaldo Cruz-Fiocruz	Yara M. Traub-Cseko Antonio J. Tempone Erich L. Telleria	ytraub@ioc.fiocruz.br trempone@ioc.fiocruz.br erichlt@ioc.fiocruz.br	*Lutzomyia longipalpis*	Jacobina, Bahia, Brazil

	Laboratorio de Bioquimica e Fisiologia de Insetos, Instituto Oswaldo Cruz-Fiocruz,	Fernando Ariel Genta	gentafernando@gmail.com genta@ioc.fiocruz.br	*Lutzomyia longipalpis*	Jacobina, Bahia, Brazil
	AV. Brasil 4365 − P26 S207, Manguinhos − Rio de Janiero, BR − PC 21040-360				

China	Xinjiang Center for Laboratory Animals,	Lifu Liao	liaolif@sina.com	*Phlebotomus wui*	Xiahe, Bachu, China
	Center for Control & Prevention in Xinjiang,			*Phlebotomus longiductus*	Kadhgar, China
	No. 380 First St. of Jianquan Urumqi Xinjiang, 830002 China				

Czech	Department of Parasitology	Petr Volf	volf@cesnet.cz	*Lutzomyia longipalpis*	Jacobina, Brazil
	Charles University,			*Lutzomyia migonei*	Brazil
	Vinicna 7, 128 44 Prague 2,			*Phlebotomus arabicus*	Israel
	Czech Republic			*Phlebotomus argentipes*	India
				*Phlebotomus duboscqi*	Senegal
				*Phlebotomus perniciosus*	Murcia, southern Spain
				*Phlebotomus papatasi*	Turkey
				*Phlebotomus orientalis*	Ethiopia
				*Phlebotomus sergenti*	Turkey
				*Phlebotomus tobbi*	Turkey
				*Sergentomyia schwetzi*	Ethiopia
				*Sergentomyia schwetzi*	Ethiopia

Egypt	Vector Biology Research Program	Hanayo Arimoto	hanayo.arimoto.mil@mail.mil	*Phlebotomus papatasi*	North Sinai, Egypt
	Navy Medical Research Unit No. 3			*Phlebotomus bergeroti*	South Sinai, Egypt
	Cairo, Egypt			*Phlebotomus langeroni*	El Agamy, Egypt
				*Phlebotomus duboscqi*	Marigat, Baringo, Kenya
					
	*Research & Training Center on Vectors of Diseases, Faculty of Science, Bldg., Ain Shams University, Abbasis	Shaaban S El Hossary Hany Ahmed Kamal Said A. Doha	shaaban92@yahoo.com doha57@yahoo.com hany63@yahoo.com	*Phlebotomus papatasi*	Alexandria, Egypt
				*Phlebotomus papatasi*	North Sinai, Egypt,
				*Phlebotomus papatasi*	South Sinai, Egypt
				*Phlebotomus sergenti*	
				*Phlebotomus bergeroti*	

France	Service de Parasitologie Maladies parasitaires	Michel Franc	m.franc@envt.fr	*Phlebotomus perniciosus*	Murcia, southern Spain
	École Nationale Vétérinaire de Toulouse				
	23 Chemin des Capelles, 31076 Toulouse Cedex 3 France				

	Service de Parasitologie-Mycologie	Pascal Delaunay	delaunay.p@chu-nice.fr	*Phlebotomus perniciosus*	Boadilla del Monte
	CHU de Nice − Hôpital de l'Archet				
	151, route Saint Antoine de Ginestière				
	CS 23078, 06202 Nice cedex 3, France				
					
Germany	Parasitus Ex, V,	Torsten J. Naucke	TJNaucke@aol.com	*Phlebotomus perniciosus*	Tours, France
	Vollberg Str. 37, 53859 Niederkassel, Germany	Susanne Lorentz	susanne.lorentz@parasitus.com	*Phlebotomus mascittii*	Baden-Wurttemburg, Germany

India	Kala Azar Medical Research Center	Puja Tiwary	tiwarypuja@gmail.com	*Phlebotomus argentipes 1*	Muzaffarpur, Bihar India
	Bagram Road, Muzaffarpur	Shakti Kumar	shaktingp@gmail.com	*Phlebotomus argentipes 2*	Muzaffarpur, Bihar India
	Bihar, India				
					
	Dept. of Vector Biology & Control, Rajendra Medical Research Institut of the Medical Sciences, Agamkuan, Patna-800007, Bihar, India	Vijay Kumar Arti Rama	vijayrnagar@hotmail.com aartirama@hotmail.com	*Phlebotomus argentipes*	Bihar, India

Italy	Instituto Superiore di Sanita, Department of Parasitology, Viale Regina Elena, 299-00161 Rome, Italy	Michele Maroli Gioia Bongiorno	michele.maroli@iss.it gioia.bongiorno@iss.it	*Phlebotomus papatasi* *Phlebotomus papatasi* *Phlebotomus perniciosus* *Phlebotomus perniciosus*	Turkey Turkey Diano Castello, Liguria, IT

Kenya	Entomology Branch, US Army Med. Res. Directorate- Kenya,	Thomas Gilbreth David Abuom	thomas.gilbreath@usamru-k.org david.abuom@usamru-.org	*Phlebotomus duboscqi*	Marigat, Baringo, Kenya
	Kisumu, Kenya				
	Unit 8900 Box 330, DPO, AE 09831				

Peru	Laboratorio de Entomologia, Centro National de Salud Publica, Instituto National de Salud Av. Defensores del Morro 2268 (Ex Huaylas) Chorillos, Lima, Peru	Edwin R. Zuñiga Mirian P. Salcedo Roberto Fernandez	edwinreqzun@gmail.com mpalominosal@gmail.com biol.rfernandez@gmail.com	*Lutzomyia verrucarum*	Mato, Caraz, Ancash, Peru

Portugal	Unidade de Entomologia Medica	Carlos Alves Pires	alvespires@ihmt.unl.pt	*Phlebotomus perniciosus*	Murcia, southern Spain
	Insituto de Hygiene e Medicina Tropical				
	R. da Jungueira no. 96, Pt-1349-008				
	Lisboa, Portugal				
					
Spain	Laboratory of Medical Entomology Institute of Health "Carlos III" Ctra. Majadahonda-Pozuelo, Km 2.3 28220 Majadahonda, Madrid, Spain	Ricardo Molina Maribel Jiménez	rmolina@isciii.es mjimenez@isciii.es	*Phlebotomus perniciosus* *Phlebotomus perniciosus* *Phlebotomus argentipes* *Phlebotomus papatasi*	Boadilla del Monte, Spain Fuenlabrada, Madrid, Spain India Zaragoza, Spain

	Departamento de Patología Animal Facultad de Veterinaria Universidad de Zaragoza C/: Miguel Servet, 177 50013 Zaragoza, Spain	J. Lucientes	jlucien@unizar.es	*Phlebotomus perniciosus* *Phlebotomus perniciosus* *Phlebotomus papatasi*	Boadilla del Monte, Spain Huesca, Spain Zaragoza, Spain

Tunisia	Laboratoire d'Écologie des Systèmes Vectoriels,	Elyes Zhioua	elyes.zhioua@gmail.com	*Phlebotomus papatasi*	Felta, Sidi Bouzid, Tunisia
	13 Place Pasteur, BP 741002 Tunis, Tunisia	Ifhem Chelbi	ifhemc2001@yahoo.fr		

Turkey	Hacettepe University, Ankara, Turkey	Bulent Alten	kaynas@hacettepe.edu.tr	*Phlebotomus papatasi*	Sanliurfa/Anatolia, Turkey
		Ozge Erisoz Kasap	ozgeeerisoz@yahoo.com	*Phlebotomus papatasi*	Konya, Anatolia, Turkey

UK	Division of Biomedical and Life Sciences,	Paul Bates	p.bates@lancaster.ac.uk	*Lutzomyia longipalpis*	Jacobina, Bahia, Bazil
	Faculty of Health and Medicine	Rod Dillon	r.dillon@lancaster.ac.uk	*Lutzomyia longipalpis*	Mato Grosso do Sul, Brazil
	Lancaster University	Gordon Hamilton	j.g.hamilton@lancaster.ac.uk	*Lutzomyia longipalpis*	Sobral 2S, Ceará, Brazil
	LA1 4YG Lanacaster LA1 4YG				
	Lancashire, UK.				

	London School of Trop. Med. and Hygiene,	Matthew Rogers	matthew.rogers@lstmh.ac.uk	*Lutzomyia longipalpis*	Jacobina, Bahia, Brazil
	Keppel Street, London WC1E 7HT, UK				

	Experimental Containment Laboratory, Kaye Group, Univ. York, Dept. Bio. and HYMS, Centre for Immunology and Infection	Paul M. Kaye Audrey Romano Johannes S. P. Doehl	paul.kaye@york.ac.uk audrey.romano@york.ac.uk johannes.doehl@york.ac.uk	*Lutzomyia longipalpis*	Jacobina, Bahia, Brazil
	Wentworth Way, Heslington, York YO10 5DD, UK				
					
USA	Laboratory of Parasitic Diseases	Kashinath Ghosh	kashinath.ghosh@nih.gov	*Phlebotomus papatasi*	Jordan
	Nat. Inst. Allergy and Infectious Diseases	David Sacks	dsacks@nih.gov	*Phlebotomus duboscqi-A*	Baraoueli District, Mali
	Nat. Inst. Health, Bethesda, MD 20892, USA			*Phlebotomus duboscqi-B*	Baraoueli District, Mali
				*Lutzomyia longipalpis*	Cavunje, Brazil

	Laboratory of Malaria and Vector Research	Claudio Meneses	menesescr@niaid.nih.gov	*Phlebotomus duboscqi*	Baraoueli District, Mali
	Nat. Inst. Health, Twinbrook Parkway,	Shaden Kamhawi	skamhawi@niaid.nih.gov	*Lutzomyia longipalpis*	Jacobina, Brazil
	Rockville, MD 20892, USA	Jesus Valenzuela	jvalenzuela@niaid.nih.gov	*Phlebotomus papatasi*	Jordan
				*Phlebotomus papatasi*	Israel

	Walter Reed Army Institute of Research, Bldg. 503, Robert Grant Avenue	Tobin Rowland	tobin.e.rowland.civ@mail.mil	*Lutzomyia longipalpis*	Jacobina
	Silver Spring, MD, 20910, USA			*Lutzomyia verrucarum*	Peru
				*Phlebotomus argentipes*	India
				*Phlebotomus arabicus*	Israel
				*Phlebotomus duboscqi*	Mali
				*Phlebotomus longicuspis*	Tunisia
				*Phlebotomus perfiliewi*	Tunisia
				*Phlebotomus papatasi*	Israel
				*Phlebotomus papatasi*	Jordan
				*Phlebotomus papatasi*	North Sinai
				*Phlebotomus papatasi*	Turkey
				*Phlebotomus perniciosus*	Italy
				*Phlebotomus perniciosus*	Tunisia
				*Phlebotomus sergenti*	Israel
				*Phlebotomus sergenti*	South Sinai
					
	Dept. Bio. Sci., University of Notre Dame,	Mary Anne McDowell	mcdowell.11@nd.edu	*Phlebotomus papatasi*	Israel
	Center for Global Health and Infectious Diseases, South Bend 46556 IN				
	343 Galvin Life Science Center, South Bend 46556 IN, USA				

	Dept. Bio., Utah State University,	Scott Bernhardt	Scott.Bernhardt@usu.edu	*Phlebotomus papatasi*	Jordan
	5305 Old Main Hill, Logan, Utah 84322, USA			*Lutzomyia longipalpis*	Jacobina, Brazil

Venezuela	*Centro Nacional de Referencia de Flebotomos de Venezuela, Seccion de Entomologia Medica − Biomed, Universidad de Carabobo, Callejón Cecilio Acosta, Urbanización Cantarrana, Las Delicias, Maracay, Venezuela	Dora Felicaiangeli	mdora@movistar.net.ve spinicrassa@yahoo.com mdora@gmail.com	*Lutzomyia pseudolongipalpis* *Lutzomyia longipalpis s.l.*	El Brasilar, Lara, Venezuela La Guardia, Nueva Esparta, Venezuela

## Collecting wild-caught sand flies for colony stock

Killick-Kendrick and Killick-Kendrick [24] noted that “The initiation of laboratory colonies of phlebotomine sand flies is far more difficult than the maintenance of already established colonies.” Indeed, acquisition of stock material from an already-existing, well-established and lab-adapted sand fly colony greatly simplifies the process of growing a new colony for research purposes. In this section, we discuss the methods for collecting sand flies live in the field and processing them for stock with which to initiate a laboratory colony.

### Species of interest

Before launching into a laborious, time-consuming and expensive project to collect wild sand flies to stock a laboratory colony, researchers should do their homework to gain a clear understanding of the behaviour and habits, as well as environmental and nutritional requirements of the species of interest. Because not all sand fly species can be collected, processed and reared using the same methods and environmental conditions, one should consider the following questions:

**Table 2:** Equipment and supplies needed to initiate a laboratory sand fly colony.

Item	U.S./International Vendor	Item No.	Comments
light traps, batteries, bulbs, wires	John Hock Co., Inc., Gainesville, FL	1012	programable operation with photo-cell on/off switch
collection nets (double-ring type)	John Hock Co., Inc., Gainesville, FL	1.45	or custom made
mouth aspirators with HEPA filter	John Hock Co., Inc., Gainesville, FL	612	or custom made
1-pint (475-ml) collection cups	Amazon.com; various sources		custom modified to make temporary holding containers
flashlights or headlamps	various sources		
Holding cages (fabric or plastic)	Precision Plastics, Beltsville, MD;		or custom-made by local fabricator
oviposition vials w/snap-on plastic lids	various local sources		custom made w/filter paper insert or plaster layer in bottom
fabric-mesh for closing vials and pots	various local sources		mesh at least 21 holes/linear cm
small plastic bottle for sugar solution	various local sources		for sugar meals for adult flies; can substitute apple slices
cotton balls	various local sources		for delivering sugar meals to adults
colored labeling tape	Fisher Scientific		for labeling collecting cups, oviposition vials, etc.
elastic bands	various local sources		assorted sizes; heavy-duty large to make holding cups
marking pens	various local sources		assorted colors
large heavy-duty plastic bags	various local sources		to envelope collecting nets and cages to maintain high humidity
plastic storage boxes	various local sources		for transporting oviposition vials and other holding containers
sponges	various local sources		to moisten and maintain humidity in plastic bags & storage boxes
field microscopes (stero and compound)	various sources		for examination and identification of sand flies
microscope slides and cover slips	Bioquip or various local sources		for examination and identification of sand flies
jeweler's forceps	Bioquip or various local sources		for handling individual sand flies
minuten nadeln/pins	Bioquip or various local sources		for making dissectin needles
wooden applicator sticks	Carolina Biologica	706865	
cooler box or insulatef bag	various local sources		to maintain cool temp. and humidity during transport
Mouth aspirator with 0.3 um HEPA filter	John Hock Co., Inc., Gainesville, FL	612	
125-ml Nalgene® straigth-sided jars/pots	Van Waters and Rogers International	16129-356	similar rigid plastic containers also work
500-ml Nalgene® straigth-sided jars/pots	Van Waters and Rogers International	16129-390	similar rigid plastic containers will work
2.54-cm plastic, perforated vent caps	ISC Plastic Parts (EU and USA)	100700060	vents for rearing pot lids
clear plastic vials with snap-on lids (10-15 ml)	United States Plastic Corp., Lima, OH	81001, 81003, 81004	for making isoline-rearing vials
1-pint (473-ml) paper cans/cup w/lids	Science Supplies	300	temporary holding/transport containers
Dental dam( 6 × 6 green-medium)	Henry Schein Inc.	H08562 or 101-0171	to close transfer opening on cardboard holding containers
Filament packing tape	Fisher Scientific Lab Equipment & Supplies	22-367375	for use in making temporary holding containers
Autoclave tape/masking tape	Fisher Scientific Lab Equipment & Supplies	11-889-14	various uses
Colored labeling tape	Fisher Scientific Lab Equipment & Supplies	varies w/color	for color coding containers
Bench top paper	Kimberly-Clark Professional, Roswell, GA	7456	for keeping bench top clean and for cage back-panel insert
Custom-made composting cabinet	Precision Plastics, Beltsville, MD		for making larva food
Cotton balls	Fisher Scientific Lab Equipment & Supplies	22-456-880	for administering sugar meals
1000 ml Erlenmeyer flask	Fisher Scientific Lab Equipment & Supplies	07-250-098	for making pipette aspirator to remove dead adults and mites
two-hole rubber stopper	Fisher Scientific Lab Equipment & Supplies	assorted sizes	for making pipette aspirator to remove dead adults and mites
5-inch glass pasteur pipettes(12.5 cm)	Fisher Scientific Lab Equipment & Supplies	13-678-20B	for pipette aspirator
Heavy-duty elastic bands	Van Waters and Rogers International	500024-286	for securing fabric screen over mouth of ovipots
Stereo microscope	various sources		to monitorimmature development
Illumiantor (fiber optice with ring guide)	LW Scientific, Lawrenceville, GA	ALPA-1502	accessory to stereo microscope
Compound microscope	various sources		for species identification, monitoring for pathogens
Reach-in environmental chamber	Caron Products, Marietta, OH		various companies sell environmental chambers
Minuten nadeln (stainless steel pins)	Bioquip Products, Inc., Rancho Dominguez, CA	1208S	for making dissecting needles
Wooden applicator sticks	Fisher Scientific Lab Equipment & Supplies	50-949-154	for making dissecting needles
Nylon oragandy fine-mesh screen	various sources		at least 21 openings per linear cm
Paper towels	Uline (multiple locations)	S-17461	
Plaster of Paris/dental plaster, 25 lb box	Henry Schein (multiple locations)	1450025HS	for lining bottoms of ovipostion.rearing pots
Portable vacuum pump	Gast (multiple international locations)		for operating vacuum aspirators in absence of built in system
Rabbit feces	Spring Valley Labs	N/A	for making larva food (check local sources)
Rabbit food	Quality Lab Products, Elkridge, MD	5-p25	any reliable local source
Custom-made sand fly holding cages	Precision Plastics, Beltsville, MD	various sizes	check local plastic fabricators
Marking pens	Office Depot/Office Max+A40	10014156	various colors as needed
Sodium hypochlorite (Clorox®)	Amazon.com		Clorox is 16.5% sodium hyphchlorite and must be dilute to 1%
Soil test sieves	Hogentogler, Columbia, MD	1309 (sieve); 8407 (pan)	No. 170 U.S.A. Standard Test Sieve (.0035 inch/90um diameter holes)
Spatulas	Lowes or Home Depot	422618	available in many stores
Sponges	Fisher Scientific Lab Equipment & Supplies	14-417	to maintain humidity in storage boxes, cages, etc.
Spray bottles and Squirt bottles	Fisher Scientific Lab Equipment & Supplies	01-189-100	for larva food prep ane egg washing procedure
Stockinette (20-cm diameter)	ProMed Inc., Louisville, KY	855801-NS508	access sleeves for cages
Plastic trays (45 × 65 × 7.5 cm; 18 × 26 × 3 in)	B & H, Photo & Electronics, New York, NY	CETP1417; CETP1114	photgraphic trays for holding oviposition/rearing pots
Plastic storage boxes (White Rubbermaid®)	Nationwide Facility Supplies, Buffalo Grove, IL	rep3506 (tub); rep 3506 (lids)	for holding oviposition/rearing pots
Tubing	Fisher Scientific Lab Equipment & Supplies	14-169-7C	for vacuum aspirators
Vacuum aspirator custom-made	Precision Plastics, Beltsville, MD		for transfer of bloodfed females to 500-ml pots
Waterpik® Oral Irrigator	Waterpik	WAT WP-60; WP	for washing eggs to control gregarines

- Why collect this particular species?
- Has the species been colonized previously? If so, what rearing methods were used?
- Where is the species endemic and abundant?
- Under what environmental conditions does the species thrive in nature (tropical, subtropical, temperate, savannah, desert, rainforest, etc.)?
- What is known about the species' habitat (microclimate, terrain, soil type, flora and fauna)?
- When is the species most abundant, or at least present in numbers sufficient to make collection worthwhile?
- Does the species undergo a seasonal diapause and, if so, when does it occur?
- What time of day or night does the species feed?
- What is known about the host-seeking/blood-feeding behaviour of the species?
- Is male lekking required to attract females to the host?
- Do the females mate before, during or after the blood meal, or all three?
- Where do females of the species feed and what are the preferred blood-meal sources?
- Where do females of the species rest during the day or night, especially after taking a blood meal?
- Researchers should also consider equipment and supplies that will be needed for the field work and to support the laboratory colony (see the example in Table 2). Additionally, it is advisable to maintain a field notebook in which to record activities, observations and findings for later reference.

### Selecting the collection site

**Figure 2:**
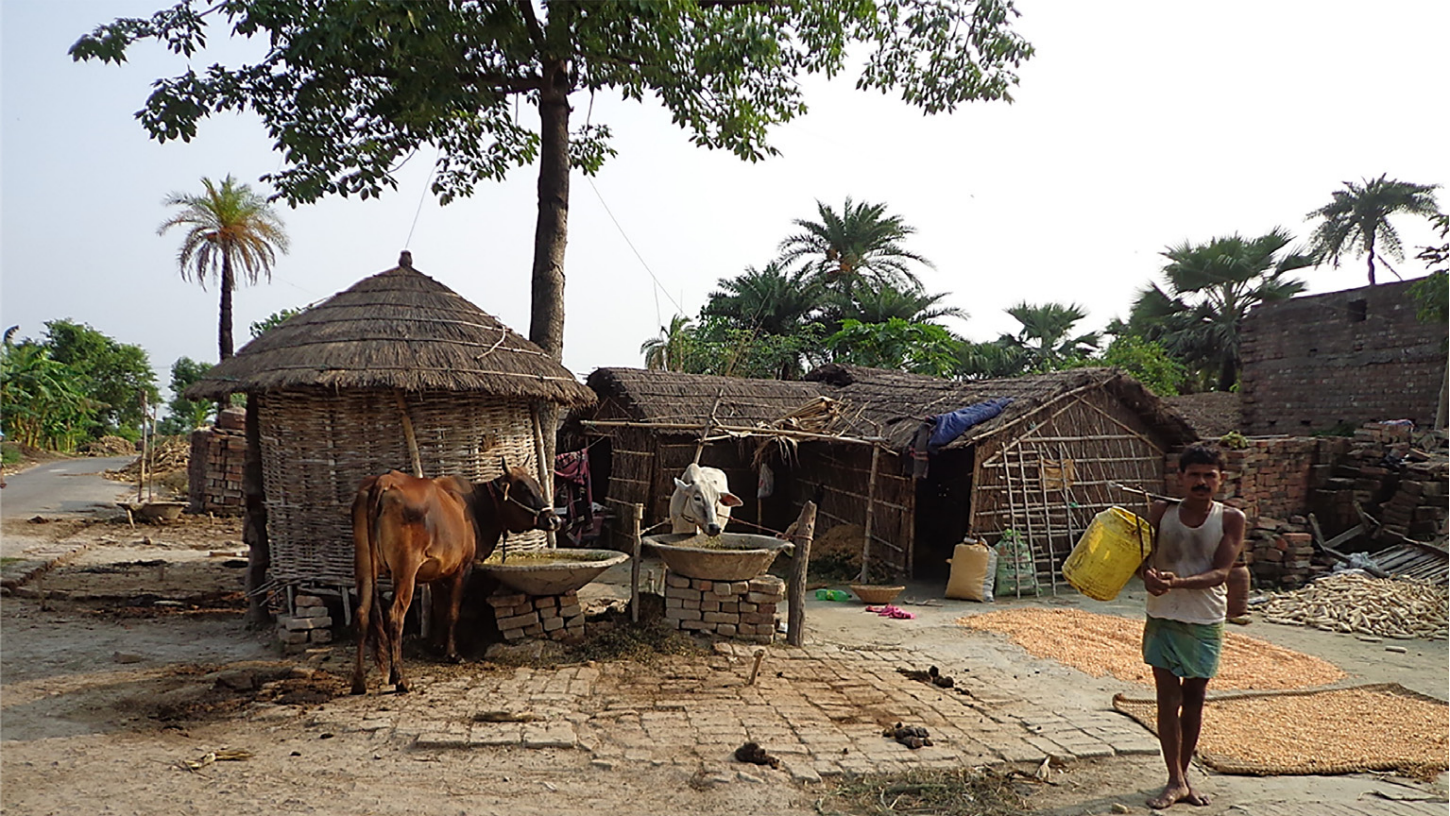
*Phlebotomus argentipes* collection site in a rural village in Muzaffarpur District, Bihar, India (Photo by P. Lawyer).

Collecting sand flies directly from the field for colony stock requires considerable planning and effort, particularly if the targeted species is in a distant country. To select a suitable collection site, one should have accurate, up-to-date information about its features to know with reasonable certainty that collection efforts will produce a sufficient number of adults to initiate a vibrant colony possessing a diverse gene pool representative of the overall population of the species (Figure 2). It is advantageous, where possible, to select a site that is monospecific in terms of the sand fly fauna, even if the population density is lower, as it obviates the task of separating non-target species. However, in most locations, more than one species may inhabit a collection site and consideration must be given as to how collected specimens will be separated by species (this will be covered in detail in paragraph 2.4). Knowledge of local seasonal changes in sand fly population density, preferably based on several years' observation, is crucial to ensure appropriate timing and to take advantage of population peaks or, particularly in temperate zones, to avoid collecting flies that are already programmed for an obligatory or facultative winter diapause. Therefore, it is also important to know the number of generations the species produces per year (seasonal dynamics) and when diapause is likely to be triggered. For example, in the Mediterranean region, sand flies complete from one to three generations per year, depending on species and locality [2]. In south-central Texas, USA, *Lutzomyia diabolica* (Hall, 1936) complete only one or two generations during the summer and then, in response to the decreasing photoperiod, begin laying diapause eggs as early as 1 September that do not hatch until April or May of the following year [40]. With such knowledge, collections can be timed to favour capturing specimens of early seasonal generations that are not likely to enter diapause. If the collection site includes human habitations, as in a rural village or in a residential area on the outskirts of a city, it is imperative to obtain approval and full cooperation of local authorities and residents. Local insecticide residual spraying (IRS) campaigns must also be considered, as they may have a significant impact on the number of flies that can be captured. One should also consider the travel distance from the collection site to the laboratory and measures to be taken to ensure survival of wild-caught specimens en route.

### Collecting methods

Various methods are used to collect sand flies alive and keep them alive for colony stock. The most commonly used involve attraction to light traps and active searches of resting sites using mouth aspirators. For a review of sand fly sampling methods, the reader is referred to [1].

#### 2.3.1 Light-traps

**Figure 3:**
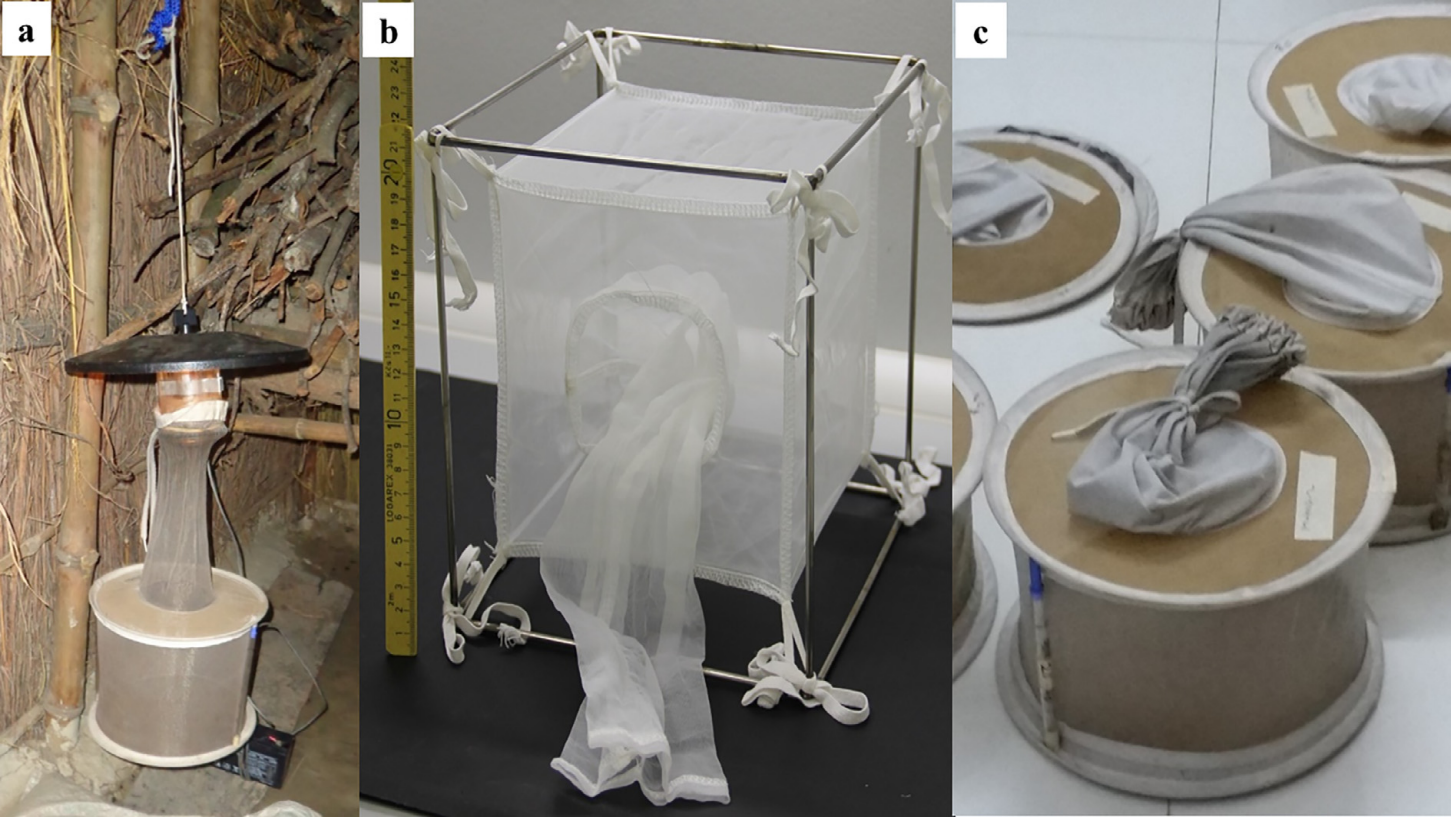
Methods for capturing sand flies and keeping them alive: a. Un-baited CDC-type light trap hung in the corner of a cattle shed (Photo by P. Lawyer); b. Fabric collection net suspended on a metal frame that can be hung from a light trap (Photo by T. Spitzova); c. Double-ring, collection nets expanded with plastic struts to prevent injury to captured flies (Photo by P. Lawyer).

CDC-type light traps (Figure 3a) are used with or without bait (CO_2_ or animal), or with lights of various colours and intensities, as some species respond better to these stimuli. For best results, traps are hung in resting or breeding sites inside human and animal dwellings, in caves or entrances to animal burrows. Light-trap collecting bags vary in shape and size. Some are especially good for keeping the flies alive after capture, such as square fabric nets suspended on metal frames (Figure 3b), or double-ring, 15-cm (6-inch) centre collection nets (John Hock Company, Inc., Gainesville, FL, USA; part #1.45), which can be expanded with struts made of sticks or disposable pipette sections to stand upright (Figure 3c).

#### Aspirators

**Figure 4:**
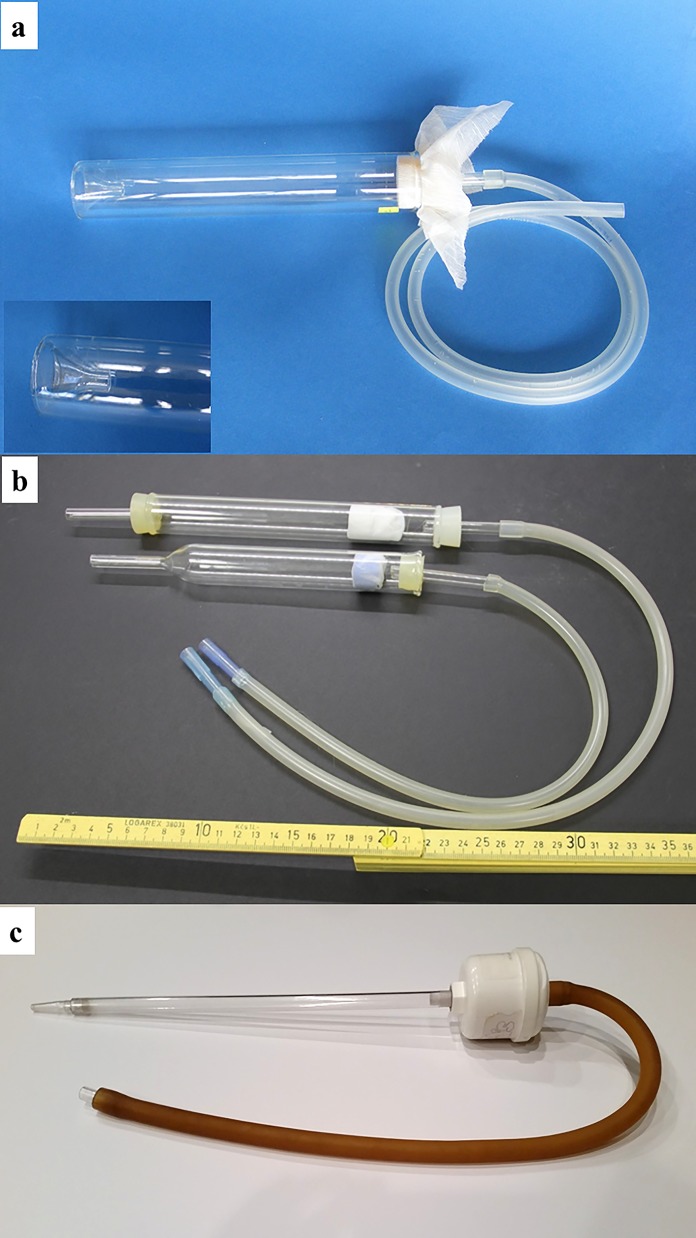
Mouth aspirators used in active searches of sand fly resting sites: a. custom-made glass aspirator (“pooter”) with inverted tip (see inset) (Photo by M. Killick-Kendrick); b. custom-made, reservoir-type glass aspirators (Photo by T. Spitzova); c. commercially available aspirator with HEPA filter (John Hock Company, Inc, Gainesville, FL, USA).

Various types of mouth aspirators (“pooters”) are used in combination with a flashlight (torch) or head lamp to actively collect flies from diurnal or nocturnal resting sites or from animals or humans used as bait (Figure 4). Some workers prefer custom-made glass aspirators or “reservoir-type” aspirators for working with large numbers of flies (Figure 4a & b) [59]. Others use commercially available aspirators (Figure 4c) consisting of a 12-mm (½-inch) diameter, 30.5-cm (12-inch) long polycarbonate tube fitted with a 0.3-micron HEPA filter (Model 612, John Hock Company, Inc., Gainesville, FL, USA). This type of aspirator is light weight, virtually unbreakable, and can be fitted easily with the modified, tapered tip of a 10-ml disposable pipette to reduce the size of the opening. Whatever the aspirator preference, the following features are essential to prevent trauma to the fragile sand flies: 1) the barrel (tube) of the aspirator should be wider than the opening to minimize the velocity of the air after it enters the aspirator; 2) the screen at the end of the barrel opposite the opening must be of sufficiently fine mesh to prevent passage of aspirated sand flies beyond the barrel (21 openings per linear cm; 52 openings per linear inch); 3) the aspirator should be fitted with some type of filter, such as a HEPA filter, to prevent inhalation of dust and debris from the collection site as well as hairs and setae from the sand flies; 4) the suction hose should be long enough to allow the collector to extend his/her reach as much as possible. When using a mouth aspirator to collect sand flies, only gentle suction should be applied to avoid injuring the flies by compressing them against the screen at the posterior end of the barrel. Some workers use hand-held, battery-operated aspirators for field work, but these are not recommended for live catches because the suction pressure usually cannot be adequately regulated to prevent injury to the flies. In many habitats, the use of mouth aspirators in combination with Shannon-type traps can be very effective (See [1] and [17]). Active catches from animal or protected-human baits using a mouth aspirator are effective for collecting host-seeking females.

#### Temporary holding containers

**Figure 5:**
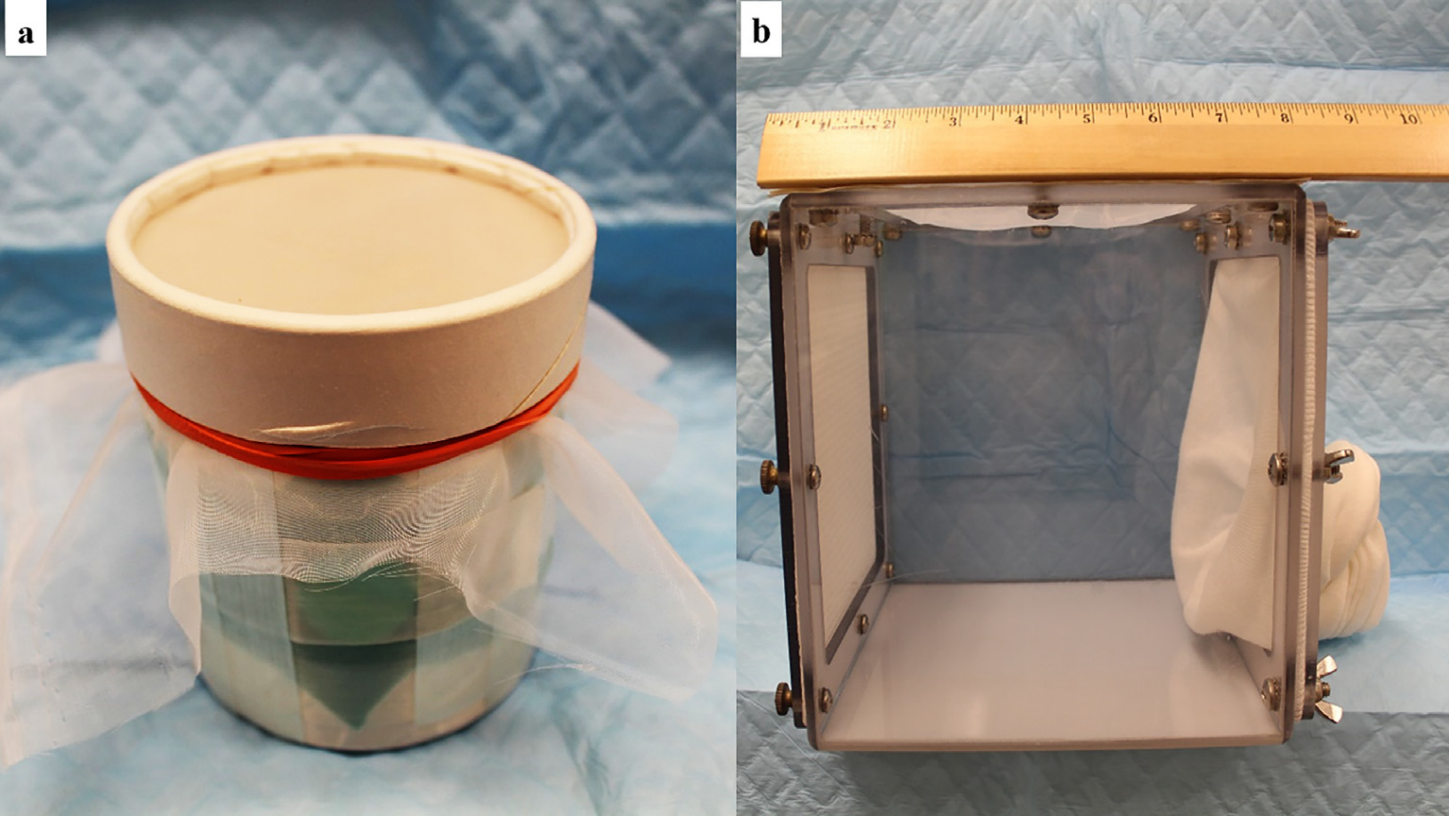
Temporary holding containers for use when collecting sand flies *via* aspirator in the field and for transporting live, unprocessed flies to the laboratory: a. modified 1-pint (473-ml paper can/cup (Photo by T. Rowland); b. small polycarbonate holding cage (Photo by T. Rowland).

When conducting resting-site collections using mouth aspirators, it is important to avoid overcrowding the flies within the aspirator. Aspirated flies can be transferred to a variety of temporary holding containers including modified cardboard cups/cans (Figure 5a), small suspended fabric-net cages (Figure 3b) or small polycarbonate cages (Figure 5b). Inexpensive, temporary collecting/holding containers such as the one shown in Figure 5a can be fashioned from 475-ml (1-pint) disposable cardboard cups/cans or similar size sturdy paper cups by cutting a 2.5-cm (1-in) hole in the side of the container for an entry portal and closing it with an escape-proof “door” made of triangular pieces of dental dam or surgical glove secured with filament packing tape. The mouth of the cup is closed with a piece of fine-mesh fabric and secured with an elastic band, tape and the lid (centre removed). (See also Appendix A.)

### Processing collected flies in the field

If travel time to the laboratory exceeds two or three hours, or if the anticipated time in the field will span one or more days, the sand flies should be transferred from the light-trap collection nets as soon as possible with a mouth aspirator to a suitable holding container. If the collections are monospecific, or if the target species can be distinguished easily from non-target species, blood-fed and gravid females (visible with the naked eye) can be selected and transferred directly *via* mouth aspirator from the collection nets to oviposition/rearing pots (see section 4.4). The size of the pot should be sufficient to accommodate the number of gravid females collected and, prior to placement of sand flies, the plaster layer in the bottom of the pot should be moistened with water (preferably distilled). Then the pots containing blood-fed and gravid flies can be put in plastic, rectangular storage boxes with tight-fitting lids or in polystyrene-foam shipping coolers. High humidity is ensured by a layer of moistened filter paper, cotton pads, sand, cloth towel or sponge in the bottom of the box. Non-gravid and un-engorged sand flies can be transferred first into a holding cage (polycarbonate or suspended fabric-net cage) and offered a blood meal on an anaesthetized mouse or hamster, or on a restrained rabbit placed inside the cage. Females can be fed on a variety of vertebrates, so the choice of animal depends on its local availability and the species of sand fly: *e.g.* *Lutzomyia longipalpis* (Lutz & Neiva, 1912) or *Phlebotomus papatasi* (Scopoli, 1786) are opportunistic and readily feed on mice, while others, like *Larroussius* or most *Adlerius* species prefer hamsters or rabbits [59]. Anaesthetized mice or hamsters are left in the cage for about one hour.

#### Sorting mixed collections

**Figure 6:**
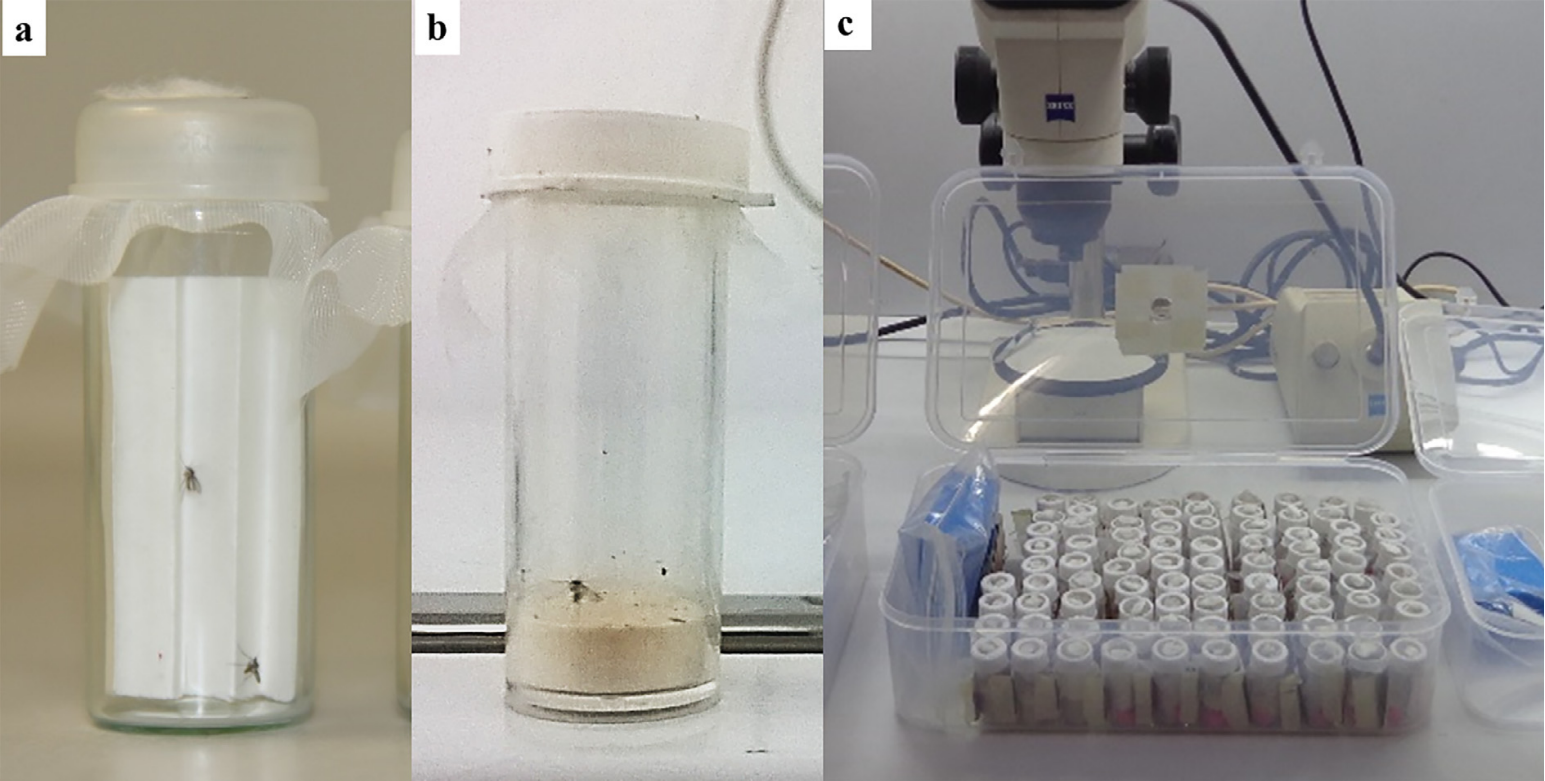
Small “isoline” vials containing individual blood-fed or gravid females: a. Glass vial with moistened filter paper as a resting/oviposition surface; b. Plastic vial with plaster of Paris in the bottom as a resting/oviposition surface; c. Isoline vials containing individual blood-fed/gravid sand flies packed in a plastic box for transport/shipping. (Photos by P. Lawyer).

More often than not, when sand flies are collected live in the field, the collected material comprises a mixture of two or more species that are difficult to separate based on external morphology. In such cases it is necessary to separate the species to ensure colonization of pure strains. This step is time-consuming and laborious but under favourable conditions can be done with wild-caught sand flies before transporting to the lab. Blood-fed or gravid females are captured/tubed individually into small vials (glass or plastic) containing an accordion-folded filter-paper insert, or into vials with a 1-2-cm layer of plaster of Paris or dental plaster in the bottom (Figure 6a & b) [15,24,38]. The mouth of each vial is closed with a square of fine-mesh fabric screen. A large hole is cut in the centre of the snap-on plastic cap forming a ring with which to secure the fabric screen. A small piece of cotton soaked in sucrose solution (30-50%) is placed on the screen top and changed daily. Each vial is labelled with the collection date and collection site of the tenant fly and the vials are stored in plastic boxes lined with moistened filter or tissue paper, or with a sponge moistened with water, preferably distilled or bottled water to avoid any adverse side effects from chlorine or other chemicals (Figure 6c). Females are stimulated to lay eggs by moistening the folded filter paper or the plaster layer inside each vial with a few drops of distilled or bottled water using a syringe with needle. After oviposition and when the parent female dies, she is removed immediately while still fresh and identified microscopically, and her species name is added to the label on the vial. The ring-type snap-on cap and fabric screen are replaced with a solid snap-on cap that is perforated with tiny pin holes to allow air to circulate in and out of the vial (make sure that the pin holes in the cap are small enough to prevent escape of the tenant larvae). The eggs are kept in the vials at a temperature similar to the natural habitat for at least 48 hrs after the date of oviposition, as during the early period of embryonic development they should not be handled. In vials with filter-paper inserts, the paper is moistened thoroughly and eggs of the same species are transferred to plaster-lined rearing pots using a fine-haired brush or by washing with distilled water [24]. In vials with a plaster layer in the bottom, the plaster is moistened and the progeny are reared to the adult stage in the vial, or eggs of the same species are pooled in plaster-lined pots as described above. The diameter of the pot used depends on the number of eggs. Identity of the progeny in each vial can then be reconfirmed microscopically when the first adult emerges. Vials, pots and cages are colour coded according to tenant species. If during routine field work such sorting is not possible, the gravid females from one locality collected on the same date can be pooled together into a rearing pot to lay eggs. Then either the live females or, preferably, their eggs are transported in the pots. After oviposition, females should be stored in ethanol for later identification. Individual separation to species can be done after the F1 generation females feed on a laboratory animal and subsequently oviposit.

#### Holding containers for transport

When considering the type of holding container to use during transport and processing, bear in mind that the less often the flies are handled, *i.e.* transferred from one holding container to another, and the less often they are disturbed to the point of flight, the less energy they will consume and the more likely they are to survive to produce eggs. The holding container must protect the flies from desiccation and excessive heat or cold. If the distance to the laboratory can be traversed within one or two hours by car, sand flies captured with light traps can be left in the expanded collection nets or suspended fabric nets (Figure 3b & c), provided there are no spiders or large insects in the nets that might eat or damage them. Depending on the travel distance, small pieces of sugar-soaked cotton may be placed on the tops of the collection nets or cages to provide a source of energy for the flies. Flies captured by mouth aspirators can be left in the cardboard collection cups (Figure 5a), or they can be transferred to a small holding cage or rearing pot for transport to the laboratory. A small (20 × 20 × 20-cm; 8 × 8 × 8-in.), custom-made polycarbonate holding cage, with a paper-insert or plaster resting surface on the back panel and a piece of sugar-soaked cotton placed on the screen top, works well for this purpose (Figure 5b). Such sturdy cages are easy to transport and provide excellent protection for the captured flies. Other, similarly modified, rigid plastic containers or rearing pots will also work. During transport, the containers should be enveloped in a plastic bag with a moist sponge or cotton pad to maintain high humidity and they must be protected from sunlight, excessive heat or cold in an insulated container, such as a polystyrene cooler or insulated bag. The collection containers and cages should be well cushioned with paper or foam pads for the flies to withstand the vibration, jostling and jarring of road travel. Damp towels draped over the holding cages, small freezer packs or waterproof bags of ice placed on the bottom of the cooler will help keep the temperature at an acceptable level. Packed securely in pots or vials inside plastic storage boxes or polystyrene-foam shipping coolers, the flies can be transported long distances to the laboratory by automobile, train or by air in checked baggage with excellent survival.

#### Import/export permits

Most countries require permits to import insects from other countries. Some, but not all countries of origin require export permits in order to take the flies out of the country. Sand fly workers must be sure to check with local authorities to determine import and export regulations pertaining to live-vector insects. Failure to comply with applicable import and export regulations may result in severe punitive consequences.

#### Tips on field-expedient use of locally available materials

When working in the field, it may be necessary to improvise with whatever materials are available. For example, depending on where the work is being done, the more expensive Nalgene^®^ pots (Nalge Nunc International, Rochester, NY, USA) may not be available or affordable, but inexpensive rigid plastic food containers readily available in most areas work well. Also, in most countries, especially in small villages where there are potters, or in markets, one can find rough, porous-clay pots of various sizes. A lip around the top of the pot facilitates covering the mouth of the pot with fabric mesh and securing it with an elastic band. Such improvised pots are excellent for transporting adult flies and for accommodating immature stages. The clay pots can be washed after use, then sterilized in an oven and used again. Also, it may be necessary while working in the field to have a local tailor or seamstress make suspended fabric-net cages to accommodate large collections of flies. Very fine and transparent cotton, nylon, or other man-made fabric mesh, can be used to make the net cages. It is very important to ask the person who is making the nets to sew them with flat double seams to avoid entrapping flies inside the creases. The new fabric cages should be washed prior to use with mild soap, such as is used for babies, or hypoallergenic liquid or powder, and rinsed thoroughly. The nets should be washed after use at each generation. In some places where stands of bamboo are common, sections of large bamboo stems have been used by sand fly workers to make temporary holding containers (MK-K). The sections are cut from the bamboo stem so that there is a closed node at the bottom; the top is open and covered with a piece of fabric mesh secured with an elastic band. A small hole is cut in the fabric mesh through which flies can be loaded. Such containers are inexpensive, are strong and offer insulation and protection for the sand flies.

## Initiating the laboratory colony

Prior to initiating a laboratory sand fly colony, whether from wild stock or from an already established colony, it is imperative that the proper infrastructure, environmental cabinets/incubators and other equipment, supplies and trained and permanent personnel are available. For specific guidance on infrastructure, see Arthropod Containment Levels (ACLs) [3]. For a list of equipment and supplies needed to initiate the laboratory colony, see the example in Table 2. Another key consideration when initiating a sand fly colony from wild-caught sand flies is to collect sufficient numbers of males and females to achieve a critical mass necessary to facilitate good social feeding behaviour and ensure a representative gene pool. The size of the critical mass may vary from species to species. Although colonies of some species have been started successfully with only a handful of egg clutches from gravid females, resulting colonies are likely to be so inbred that they bear little resemblance to the natural population and may collapse after several generations due to genetic bottle necks. It is best to start with as many gravid females as possible (at least 30, preferably hundreds) and, if feasible, infuse the colony frequently with new stock from the original collection site until it is self-sustaining and well enough established to ensure a representative, healthy and diverse gene pool. However, a word of caution: every infusion of wild stock represents a risk of contaminating the colony with pathogens such as gregarines or even viruses.

### Overview of sand fly biology

**Figure 7:**
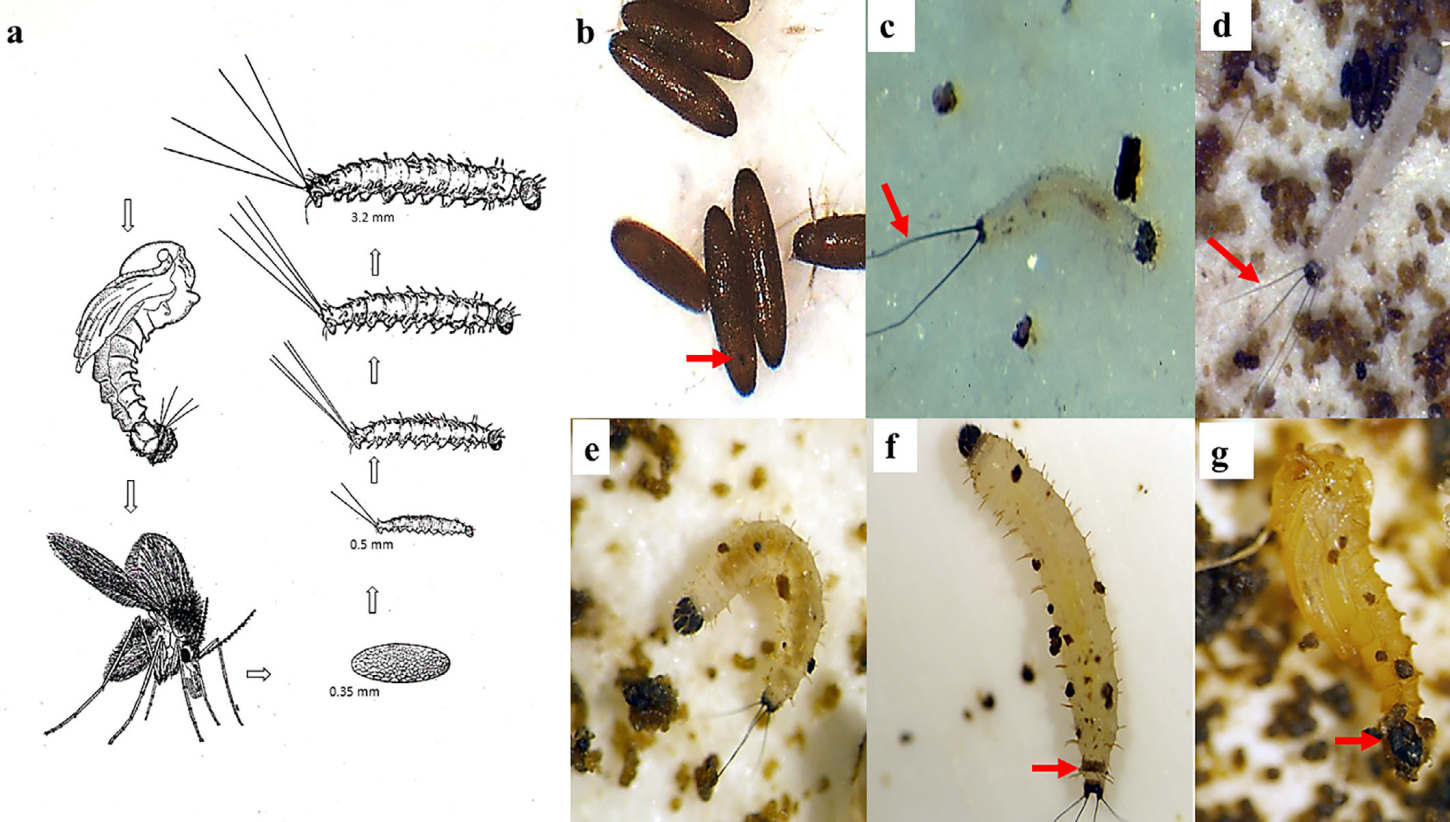
Sand fly life cycle: a. Life stages of *Lu. diabolica* showing relative sizes (Not drawn to scale. Artwork by M. Duncan and H. Muñoz); b. Eggs – arrow points to “burster spot”; c. 1^st^ instar larva – arrow points to two caudal setae; d. 2^nd^ instar larva – arrow points to 4 caudal setae; e. 3^rd^ instar larva; f. 4^th^ instar larva – arrow points to dorsal anal plate; g. Pupa – arrow points to 4^th^ instar exuvium at the caudal end of the puparium (Photos not to scale; Photos by E. Rowton and T. Rowland).

It is imperative that workers who are anticipating or are in the process of initiating a laboratory sand fly colony become familiar with sand fly biology and morphology so that they can recognize each immature life stage and accurately chronicle development through adult emergence (Figure 7a). Unlike mosquitoes and other biting Diptera, sand flies are strictly terrestrial in all stages of their development and their life cycle is of relatively long duration, with generation times of one to three months depending on rearing conditions such as temperature, humidity and nutrition. Most sand flies require a warm, humid environment to thrive. However, some are adapted to cooler or drier conditions such as *Lutzomyia verucarrum* (Townsend, 1913) and *Lutzomyia peruensis* Shannon, 1929, two species that occur in the high Andes Mountains of Peru, and *Phlebotomus orientalis* Parrot, 1936 from Sudan. Both male and female sand flies require carbohydrates (sugars) as an energy source, which they obtain from floral nectars and other plant juices, as well has from aphid honeydew [8,29]. Only female sand flies bite and require blood meals to produce eggs. Sand flies exhibit complete metamorphosis. The immature stages include egg, larva (four instars) and pupa. (See also [35].)

#### Egg stage

Sand fly eggs are small (0.3-0.5 mm long and 0.1-0.15 mm wide), elliptical in shape and range in colour from white, when freshly deposited, to brown or black (Figure 7b). The surface of the egg exhibits chorionic sculpturing of ridges and protuberances that form distinct patterns unique for each species or species complex [15,16]. A dark spot (“burster spot”) appears at the anterior end of the egg approximately 24 hours before hatching (Figure 7b, arrow). This spot is actually a spine on the apex of the head capsule of the first instar larva with which the first instar scores the inside of the egg capsule, weakening it until it bursts open, facilitating the tenant larva's emergence. Female sand flies usually lay between 30 and 70 eggs depending on the species, size and nature of the previous blood meal, larval diet, and other factors [59,65]. Benkova and Volf reported that for *P. papatasi* maintained at various temperatures, the maximum number of eggs laid by a single female was 115 [7]. Some sand fly species oviposit their first batch of eggs autogenously (without having taken a blood meal) [43]. However, for subsequent gonotrophic cycles, the females must usually take a blood meal. Eggs are deposited singly or in clusters in moist, protected places such as rock crevices, bases of trees, tree holes, under leaf litter on the forest floor, animal burrows, animal shelters and similar microhabitats. For some colonized species, roughening or making grooves in the plaster of Paris oviposition surface may help increase egg laying. Typically, the eggs hatch within 6-11 days post blood meal [59] but some may have an extended incubation period of 30 days or more, especially if exposed to unfavourable conditions.

#### Larva stage

Sand fly larvae range in size and colour but are characterized as small and caterpillar-like. The colour of the larva varies by species but generally ranges from white to grey. Larva size increases with each instar (Figure 7a). First instar larvae are tiny (< 1 mm in length; Figure 7c). The head capsule is dark (except within the first few hours after hatching when it is white or light grey until it hardens). Lateral setae are present but extremely small. Two caudal setae are present and visible. Second instars are larger than first instars (< 2 mm) and bear four caudal setae (Figure 7d). Third instars are larger and more robust than second instars (< 3 mm) and also bear four caudal setae (Figure 7e). Fourth instars are even larger (< 4 mm) and likewise have four caudal setae; lateral setae are more pronounced. A distinguishing characteristic of the fourth instar is a heavily sclerotized dorsal anal plate (Figure 7f). Size may also depend on age, nutrition and species; some fourth instars may grow up to 4 mm in length. *Phlebotomus tobbi* Adler, Theodor and Lourie, 1930, appears to be an exception in terms of the caudal setae present in that each of the four instars bears only two [34].

#### Pupa stage

At the end the fourth stadium, the fourth instar stops feeding and evacuates its gut and the larva becomes opaque white in colour. As transformation to pupa begins, the anterior third of the lava body becomes swollen; this is called the “pre-pupa”. Within the next 24 hours, pupation is completed. The pupa is 3-4 mm long and resembles a butterfly's chrysalis (Figure 7g). The collapsed exuvium (cast-off skin) of the fourth instar can be seen at the caudal end of the puparium. Pupae in early development appear whitish then turn orange or reddish brown to black as eclosion nears.

#### Adults

Adult sand flies are distinct in that they have hairy bodies and wings are held at 45-degree angles above the body when at rest (Figures 1 and 7a). Only the female sand fly requires a blood meal, which is used as a protein source to produce eggs. Sand flies are generally considered to be weak flyers with flight patterns consisting of short hops. However, some studies have shown them capable of flying relatively long distances (several km), sometimes against the wind, in two or three nights, depending on prevailing air currents and other environmental factors [10,32]. Sand flies are pool feeders with mouthparts consisting of six bladelike stylets. Male sand flies have clasping structures on the tip of the abdomen that are used for mating.

### Isoline rearing and species identification

As mentioned in paragraph 2.4.1, if field-collected material is a mixture of two or more species, it is necessary to separate the species to ensure colonization of pure strains. This is done through a process known as isoline rearing in which blood-fed and gravid females are captured individually into small (10-15 ml) rearing vials (Figure 6) and are allowed to oviposit. Then, the progeny are reared to adulthood in single broods. Experience has revealed the following advantages of isoline rearing: 1) Females set up in isoline vials, as opposed to larger oviposition containers, are more sedentary and expend less energy flying around, resulting in better survival and higher egg production; 2) after the parent female dies, she can be removed and identified to species, thus also identifying her progeny and enabling separation of species; 3) rearing the progeny of each parent female separately facilitates life-table studies to chronicle immature development and determine fecundity, productivity and overall generation time.

### Life-table studies

As previously mentioned, initiating laboratory sand fly colonies from wild stock is difficult, time-consuming and uncertain until the developmental parameters of the species are known. These parameters are obtained through daily observation of isoline-progeny development of blood-fed females. For life-table studies, it is recommended that a sampling of at least 30 isoline broods of each species be examined daily for one or more generations (preferably F2 or later) to mark significant developmental events such as egg hatch, moult from one instar to the next, pupation and adult emergence. These data are recorded on a life-table data collection sheet and summarized to produce a stage-specific life-table (Table 3). Information derived from life-table studies facilitates predicting when a particular life stage will appear, as well as revealing generation time, fecundity, productivity and sex ratio of the colony. Isoline broods with similar collection dates that are in excess of those needed for collecting life-table data can be pooled (after the appearance of the second instar) in larger rearing containers such as 125- or 500-ml rearing pots for mass rearing.

**Table 3:** Life-table attributes of three Tunisian species. Sand flies collected in a light trap from a site in Tunisia were blood fed and transferred to an oviposition/rearing pot where they laid eggs. As several species were known to occur at this particular site, the F1 progeny of the parent adults were reared together. The F1 females were blood fed and transferred individually to oviposition/rearing vials and F2 progeny were reared as isolines. The parent females were identified postmortem and their progeny were separated by species and their development chronicled as shown below.

Life Table Attributes (Second Generation)	Phlebotomus perniciosus	Phlebotomus longicuspis	Phlebotomus perfiliewi
Tunisian Sand Flies (26 °C, 80% RH)	(PRTN)	(PLTN)	(PFTN)
			
Developmental Time in Days	Mean (Standard Deviation)	Mean (Standard Deviation)	Mean (Standard Deviation)
			
Blood Meal to Oviposition	6(2.16)	8(1.86)	9(2.12)
Oviposition to Egg Hatching	7(1.52)	8(5.13)	5(1.66)
Egg Hatching to 2nd Instar (1st Stadium)	6(1.41)	6(5.78)	7(2.16)
2nd to 3rd Instar (2nd Stadium)	4(1.28)	4(2.89)	5(1.77)
3rd to 4th Instar (3rd Stadium)	5(3.06)	5(2.89)	6(2.80)
4th to Pupa (4th Stadium)	8(1.88)	8(4.18)	7(4.50)
Pupa to 1st Adult (Pupal Stage)	10(1.64)	9(3.35)	9(2.94)
Oviposition to 1st Adult	40(3.31)	40(4.20)	39(6.86)
Oviposition to 1st Male	40(3.33)	39(3.71)	40(6.50)
Oviposition to 1st Female	42(3.31)	43(4.50)	42(4.65)
Protandry	2(1.91)	4(2.59)	4(3.72)
Mean Number of Days of Male Emergence	5(2.17)	6(2.83)	5(2.70)
Mean Number of Days of Female Emergence	7(2.29)	5(2.91)	5(2.92)
Mean Number of Days to Adult Emergence	6(2.37)	5(2.87)	5(2.77)
Mean Generation Time (BM to 1st Adult)	46	47	48

Fecundity/Productivity			
Mean Number of Eggs/Female	34(12.85)	29(13.24)	32(9.50)
Range	2-57	7-66	12-49
Number of Fertile Egg Batches	53	44	24
Number of Egg Batches Producing Adults	50	37	16
Number of Adults Emerged (#M,#F)	1048(405,643)	625(308,317)	232(112,120)
Sex Ratio (Males/Total Adults)	0.39	0.49	0.48
Rate of Increase (Females/#Egg Batches)	13	9	8

## Colony Maintenance Procedures

Sand fly colony maintenance is complex, tedious and time-consuming, even with established colonies. Tasks and procedures must be accomplished in a timely and accurate manner. Failure to do so will be detrimental to the colony. A weekly colony data log sheet is used to ensure that colony maintenance tasks are accomplished each day according to the established routine. Also recorded on the sheet are daily temperature and humidity readings, the number of blood-fed females captured after each feeding, as well as the number of flies (males and females) removed from the colony for research purposes. See the example in Appendix B. Such data give researchers a clear picture of colony size, rate of growth, the impact of research demands and the overall health of the colony. Log sheets can be modified consistent with the routine and needs of each laboratory. Managing sand fly production against sand fly usage is critical. Working sand fly colonies must be large enough to support the demands of ongoing research without negatively impacting colony health and robustness. If the research demand for sand flies exceeds production, the colony population will decline. Therefore, the colony population must be sustained above a healthy threshold so that it does not decline. Experience has shown that the number of female sand flies removed from the colony for research in a given week should not exceed one-third of the number of females produced in the same week.

### Rearing Conditions

**Figure 8:**
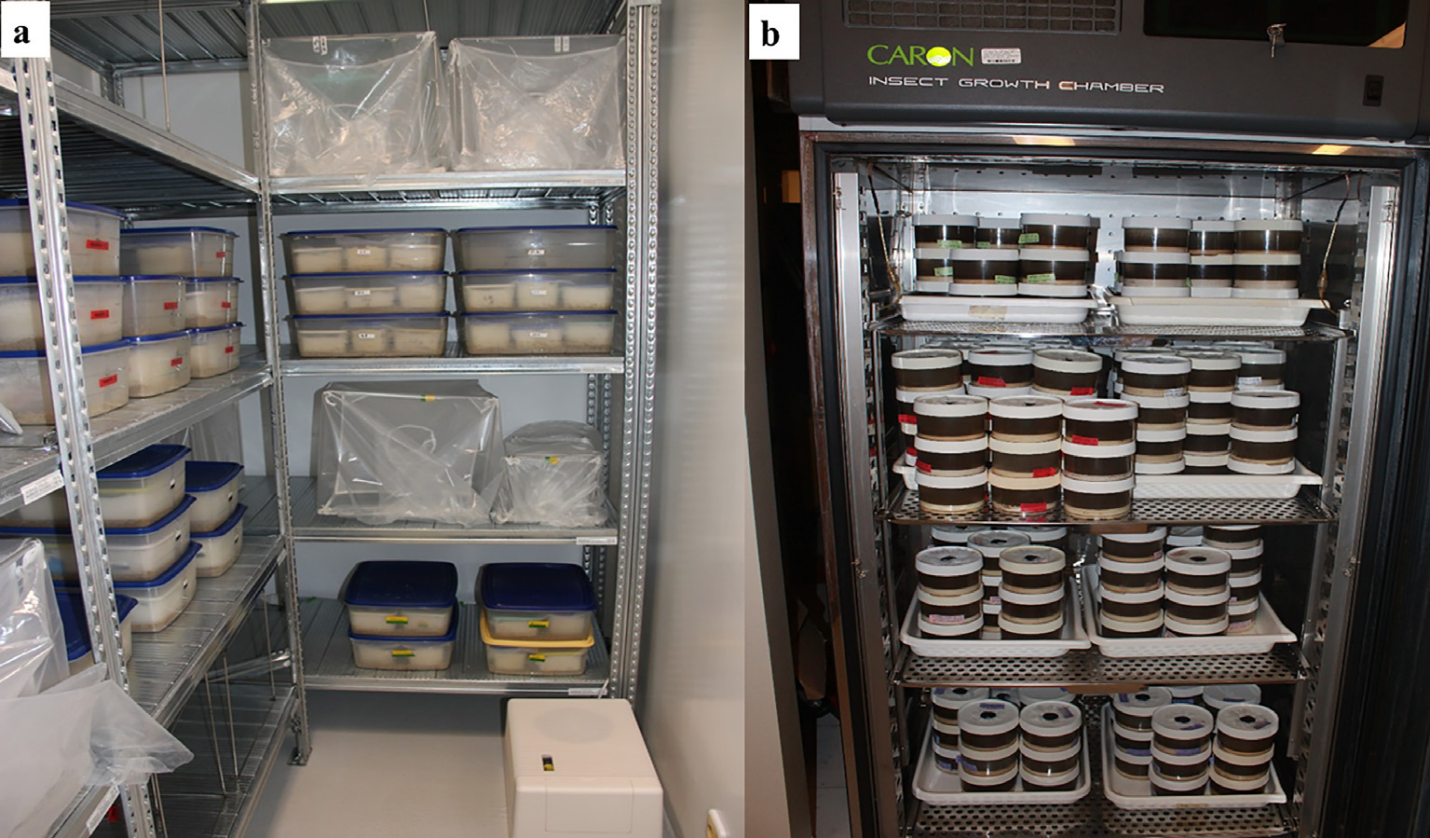
Facilities for housing sand fly colonies under prescribed temperature, humidity and light conditions: a. walk-in environmental room (Photo by T. Spitzova); b. reach-in environmental cabinet (Photo by E. Rowton).

In general, sand fly colonies are maintained in reach-in environmental cabinets or in walk-in environmental rooms (Figure 8) at temperatures between 24−28°C and 70-80% relative humidity (RH). However, environmental parameters may vary depending on the species or life stage. For instance, a *Lu. verrucarum* colony that originated from the Andes mountain region of Peru is maintained at 22°C because it does not thrive at higher temperatures. Adults of a *Phlebotomus argentipes* Annandale & Brunette, 1908 colony, originating from India, do best at 26-28°C and RH higher than 80%. Adults of tropical species such as *Lu. longipalpis* survive best at 80% RH, whereas adults of desert/savannah species such as *P. papatasi* or *Phlebotomus duboscqi* Neveu-Lemaire, 1906, do well at 70-75% RH. Immature stages of these species mature faster at 26°C and the adults survive longer at 25°C. Many incubators and environmental rooms have a light-cycle function that can be set to approximate the photoperiod that occurs in the field, *i.e.* 12 hr light:12 hr dark; 14 hr light:10 hr dark; 16 hr light: 8 hr dark, etc. This is not always a critical factor and most colonies can be maintained in incubators that do not have a light cycle function with no noticeable differences in feeding behaviour, egg production or larval development. However, there are exceptions: One of the authors (MK-K) observed that *P. orientalis* from Sudan feed well in total darkness, but do better if kept under a 12 hr light: 12 hr dark light cycle. Where incubators or environmental rooms are not available, cages and pots can often be maintained at stable room temperatures (∼24-26°C) on bench tops or tables when enveloped in plastic bags with a wet sponge to maintain high humidity.

### Adult holding and mating cages

**Figure 9:**
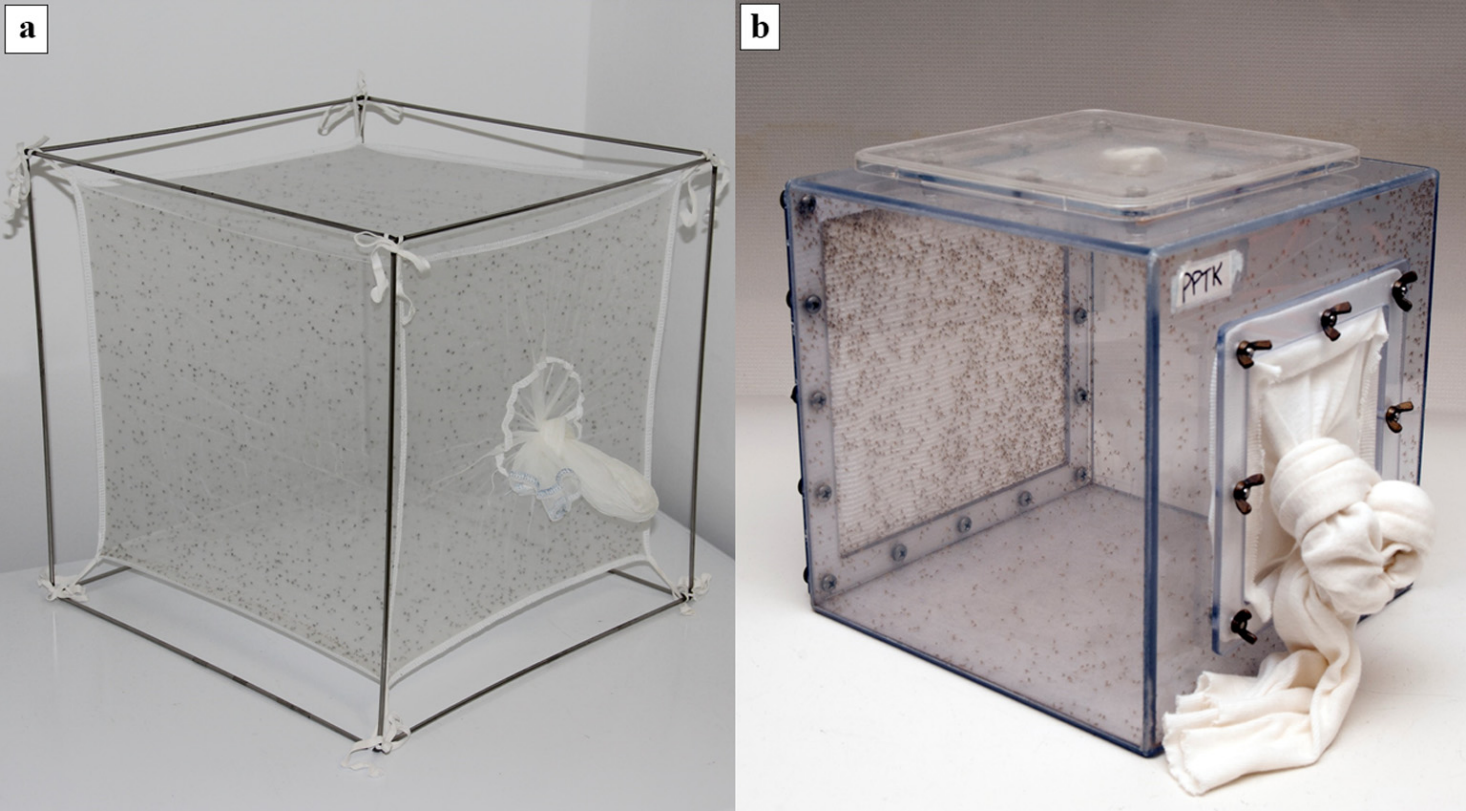
a. Large (30 × 30 × 30 cm) fabric-net adult holding cage suspended on a metal frame (Photo by T. Spitzova); b. Custom-made polycarbonate adult holding cage (30 × 30 × 30 cm) with a paper-insert resting surface on the back panel used for holding live flies in the field and during transport. A piece of sugar-soaked cotton is placed on the screen top as an energy source for the flies (Photo by E. Rowton).

A variety of cages have been devised to contain adult sand flies. Two of the most commonly used are fabric-net cages suspended on wire frames and custom-made polycarbonate cages (Figure 9).

#### Fabric-net cages

These cages can be custom made or purchased commercially in a variety of sizes depending on the preference of the researcher and the requirements of the sand fly species (Figure 3b and Figure 9a). The fabric-nets are suspended on stainless steel, aluminium or plastic frames and can be machine washed after each adult generation with hypoallergenic detergent without optical brighteners or odorants. These cages are inexpensive and light weight. The fabric netting provides an excellent resting surface for the flies and good visibility. Sugar meals can be provided by placing pieces of cotton soaked in sucrose solution on the top of the cage or in a Petri dish placed inside the cage. Anesthetized animals used as blood-meal sources can be placed on the top of the cage for the flies to feed on through the netting or they can be placed inside on the floor of the cage.

#### Polycarbonate cages

Custom-made polycarbonate cages can be lined with a thin layer of plaster on the bottom and back panel, or fitted with a removable back panel on the interior side of which is inserted a sheet of absorbent bench-top paper that provides an excellent vertical resting surface for the flies (Figure 5b and Figure 9b). The removable screen on top of the cage allows for good ventilation and provides a surface on which a sugar-soaked piece of cotton can be placed and through which the flies can obtain sugar meals. A 20 × 20 cm (8 × 8 in) culture plate placed over the screen top prevents the sugar-soaked cotton from drying too rapidly. These sturdy cages are very durable (some have been used for more than 10 years) and can be washed with mild detergent and sanitized with 70% ethanol after each adult generation. The screen tops, sleeves and backing can also be washed and/or replaced between adult generations. Cage sizes range from to 20 × 20 × 20 cm- (8 × 8 × 8 in) to 30.5 × 30.5 × 30.5 cm (1 × 1 × 1 ft), depending on the size of the colony.

### Adults

#### Mating, sugar feeding and blood feeding

Upon emergence, adult flies are released into holding/mating cages (Figure 9). A 30 × 30 × 30-cm (1 × 1 × 1-ft) cage can easily accommodate up to 3,000 flies without significant overcrowding. Balls of cotton saturated with 30-50% sucrose in water are placed on the screen tops of the cages to provide a sugar meal for flight energy and longevity. Apple slices placed on the top screens or in Petri dishes placed inside the cages also work well. Sugar sources may vary depending on local availability. Mating occurs before, during and after feeding (in most) according to the species and commences shortly after the females emerge. Three to five days after emergence, the females are ready to take a blood meal. Various blood-meal sources can be used depending on availability of source animals and the feeding preference of the flies. In the initial stages of colony establishment, the flies may refuse to feed on anesthetized mice or hamsters and may prefer a larger animal such as an anesthetized nude Guinea pig or a restrained rabbit. Some laboratories use restrained chickens with good success, but they are rather messy. Others use artificial membrane feeders loaded with rabbit or human blood, but the feeding success is considerably lower than with live animals [13,45,64]. It may be necessary to try several blood-meal sources and feeding times before an acceptable combination is found. Sand fly workers should consult with local institutional veterinary personnel regarding the appropriate anaesthesia regimens for animals used as blood meal sources for the sand flies. When using live animals, an animal-use protocol approved by the local institutional animal ethics committee is essential (the titles of such committees vary from country to country, *i.e.* Institutional Animal Care and Use Committee in the U.S.; Institutional Committee on the Ethics of Laboratory Experiments in the Czech Republic). One day prior to blood feeding, the sugar pads are removed from the cage tops and the flies are starved for 24 hours, after which anesthetized animals such as mice or hamsters, eyes protected from bites with ophthalmic ointment or wet cotton wool, are placed on their backs inside the cage to provide a blood meal. When the animals recover from the anaesthesia, they are removed immediately from the cage to prevent soiling or damaging the cage and are returned to housing. Consult the pertinent animal-use protocol for the number of times and frequency at which a particular animal can be used to feed the sand flies. The blood-fed flies are left in the cage for at least 24 hours post-feeding to allow time for diuresis and for the fragile peritrophic membrane that surrounds the blood meal to harden. This also allows for further mating. For many species, unnecessary handling prior to 24 hours may cause the peritrophic membrane to rupture, ultimately killing the fly. The larger the stock of wild-caught flies available to initiate a colony, the greater the production of F1-generation adults, and the sooner a critical mass of males and females can be achieved to stimulate optimum mating and feeding behaviour, and the sooner the colony will become self-sustaining. Expansion of the colony can be accelerated by feeding the flies at least three times per week.

#### Oviposition

As different types of pots and procedures are used with equal success for oviposition and rearing, it seems appropriate to describe two systems that are most commonly used and let the readers decide which they prefer based on species, number of colonies, available facilities, resources and manpower.

##### System 1. Charles University

**Figure 10:**
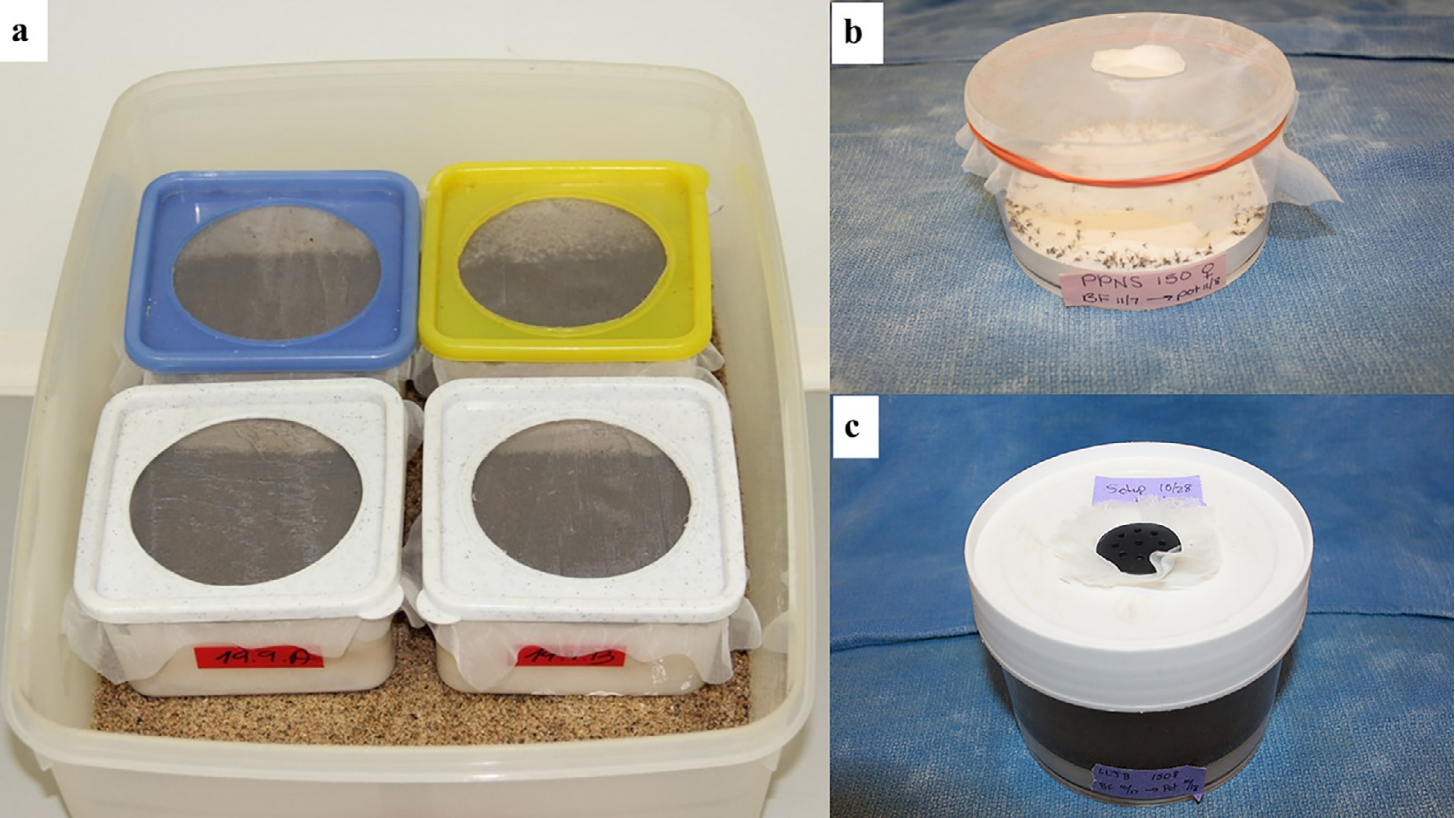
Oviposition/rearing containers: a. Rigid, modified food containers lined on the bottoms and sides with plaster of Paris (Photo by T. Spitzova); b. 500-ml Nalgene^®^ ovipositon container with plaster layer in the bottom (Photo by T. Rowland); c. 500-ml rearing container with vented solid lid (Photo by T. Rowland).

[59] (Based on the methods developed by MK-K at the Imperial College, Ascot, U.K.): Oviposition/rearing pots (ovipots) are made from solid, clear plastic containers. A large hole is cut in the bottom of the container and the interior walls are roughened with sandpaper. The container is then placed on a smooth surface, such as a glass plate, and the bottom is filled with a 1-cm layer of white plaster of Paris. After the layer of plaster has hardened, a second very thin layer is plastered on the roughened interior walls. The plaster helps maintain the humidity in the pot and provides a resting surface without water condensation. The pot is closed with fine gauze and snap-on or screw-on lid, the centre of which is cut out (Figure 10a). The gauze should be fine enough to prevent escape of larvae (at least 21 openings per liner cm; 52 openings per linear inch). Females are put in the pot (moistened with distilled water) *via* a small slit in the gauze using a mouth aspirator; the slit then is plugged with a piece of cotton. Three sizes of pots are used, the smallest (6 cm diameter) for up to 20 gravid females, and the largest (14 cm diameter) for 100-150 gravid females. Cotton balls soaked in 50% sugar are placed on the screen tops of the loaded pots as an energy source and the pots are stored in plastic boxes inside an environmental incubator at 26°C and 75-80% RH. Sugar meals are replaced three times per week. Importantly, females are left in the cage until defecation and only then are they transferred to moistened pots to lay eggs. This results in very fast and synchronous oviposition and reduces fungal growth. In some colonies, fed females are left undisturbed in a large cage for 24 hrs and then transferred to a small cage (20 × 20 × 20 cm) for defecation. When they are ready to lay eggs (for most species 5-6 days post-blood feeding), they are transferred to moist oviposition pots with an aspirator. This “two-step” procedure prevents the early contamination of rearing pots by fungi. Selecting blood-fed females is laborious but it is necessary in colonies where only a proportion of females take a blood meal. For large colonies with high feeding rates (> 90%), a “one step” procedure is frequently used. Females are left to defecate in the first cage and then all females (together with males) are transferred directly into the breeding pot. Such a procedure is advised especially for *Lu. longipalpis*, where the feeding rate may reach almost 100% and the dark colour of adults makes the selection of blood-fed females difficult.

##### System 2. Walter Reed Army Institute of Research

**Figure 11:**
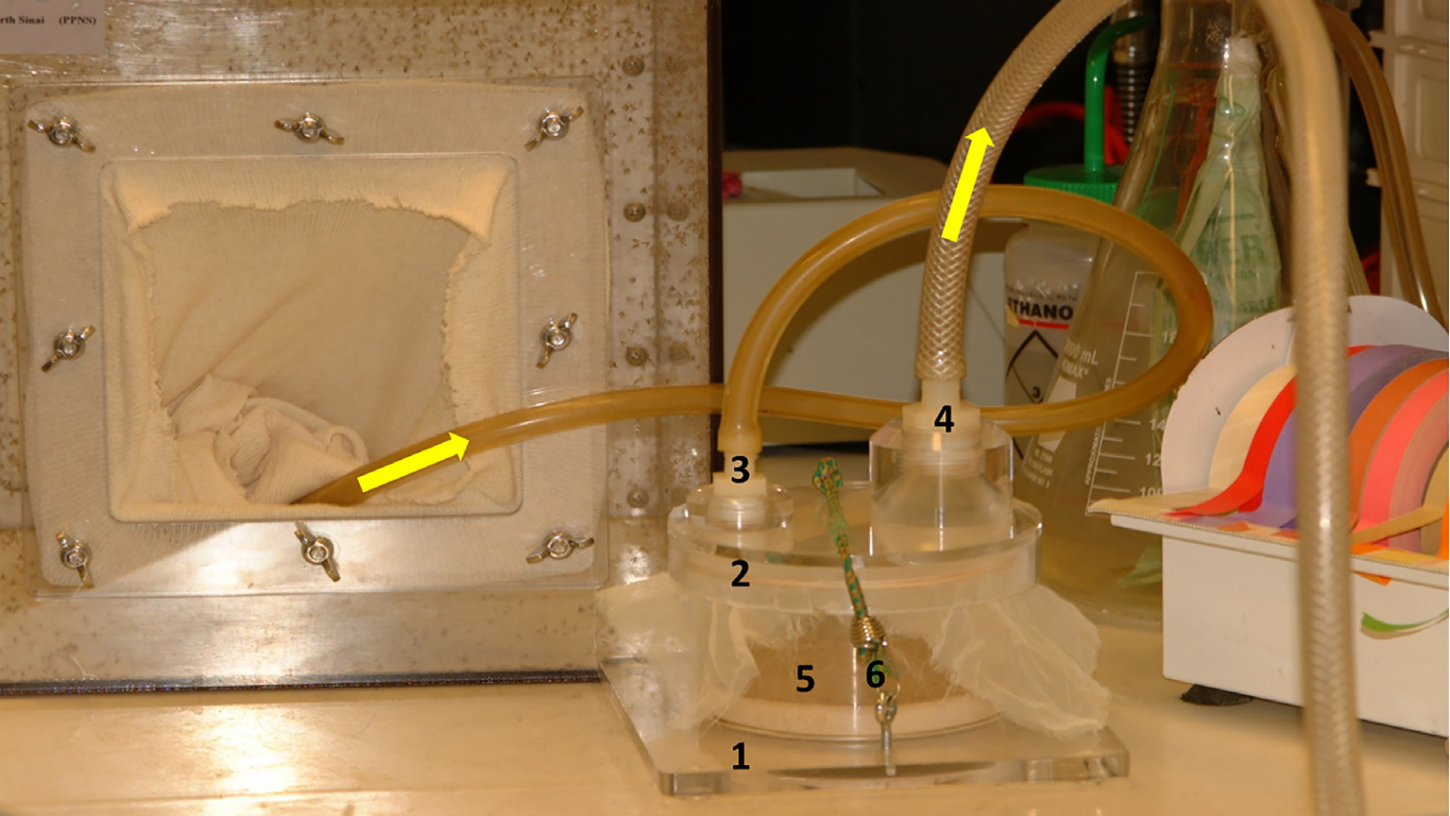
Vacuum-powered aspirator (Precision Plastics, Beltsville, MD, USA) for rapid transfer of large numbers of blood-fed flies from holding cages to ovipots: 1. Holding plate; 2. Aspirator lid with attached transfer and vacuum hoses; 3. Transfer port and transfer hose; 4. Vacuum port and vacuum hose; 5. A 500-ml ovipot nested in the depression of the holding plate; 6. Elastic securing cord hooked into screw eye ring on the holding plate. Arrows indicate the direction of air flow. (Photo by T. Rowland).

**Figure 12:**
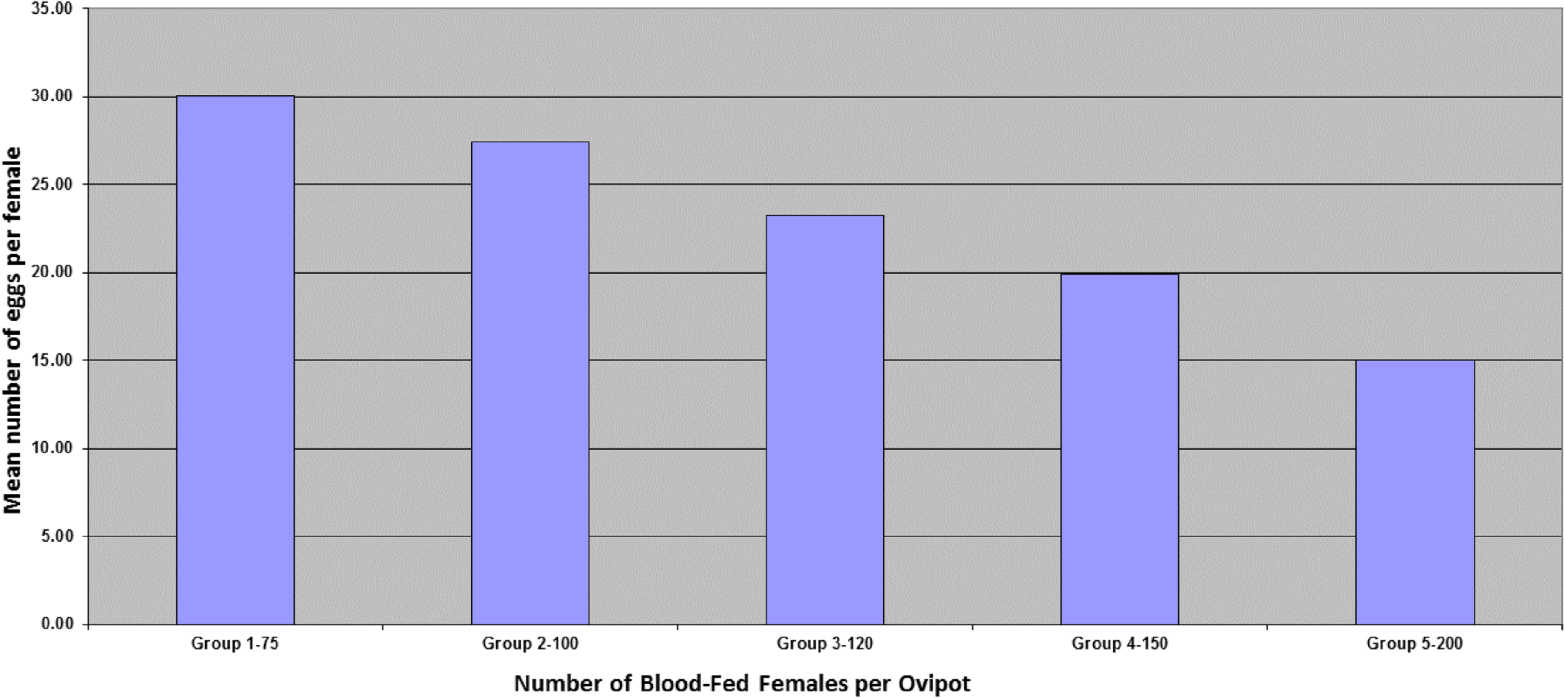
Graph showing the impact of increasing the number of blood-fed females from 75 to 200 per ovipot.

[39,49]. Ovipots are made from 125-ml or 500-ml straight-sided polypropylene jars (Nalge Company, Rochester, NY, USA) modified by drilling 2.5-cm (1-in) diameter holes in the bottoms (three holes for 125-ml pots and six for 500-ml pots). The pots are then placed on a sheet of aluminium foil on a smooth counter top and plaster of Paris is poured into each to a level of approximately 2 cm, thus providing a porous oviposition surface that can be saturated from the bottom up (Figure 10b). The open mouth of each pot is covered with a fine-mesh screen (21 openings per liner cm; 52 openings per linear in) and secured with a heavy-duty elastic band (Figure 10b). For small colonies, blood-engorged females are transferred from mating/feeding cages to dry ovipots with a mouth aspirator through a small slit in the screen cover. However, for large colonies with several hundred blood-engorged females per feed, a custom-made vacuum aspirator (Figure 11) is used for rapid transfer of the flies to dry ovipots. The aspirator is made of polycarbonate plastic and consists of a holding plate (1) and a lid (2). It has two ports, a small one for the transfer hose (3) and a larger one for the vacuum hose (4). The transfer hose is attached to the nipple of the transfer port, which has an extension that protrudes from the underside of the aspirator lid through a slit in the screen covering of the pot. The vacuum hose is attached to a nipple that protrudes from the larger port. A dry 500-ml ovipot (5) fits snuggly in a depression in the holding plate and the lid of the aspirator is secured over the top of the pot with an elastic cord (6) that hooks into screw eye rings on the holding plate, making a tight seal around the mouth of the pot. On the upper side of the aspirator lid, the flexible transfer hose extends for about one meter and has a tapered tip modified from a plastic disposable pipette. The user inserts the tipped end into the cage to capture the blood-fed flies. The aspirator is used under low vacuum pressure and is less traumatizing and less injurious to the flies than a mouth aspirator and can transfer several hundred blood-fed flies to pots in just a few minutes. After the flies are transferred, the aspirator is removed and the slit in the screen is either plugged with cotton or is slid over the rim of the pot below the elastic band that secures the screen. The number of flies per ovipot depends on the species and the size of the pot, *i.e.* up to 50 engorged females and about 20 males in the smaller-sized pot (125-ml) and 100-200 females and about 50 males in the larger pot (500-ml). Cotton balls soaked in 30%-50% sugar are placed on the screen tops of the loaded pots as an energy source and the pots are stored in plastic boxes inside an environmental incubator at 26°C and 75-80% RH. Sugar meals are replaced every other day. After five days, the plaster in the bottoms of the pots is saturated by placing the pots in a tray with water. Saturating the plaster stimulates almost immediate and synchronous oviposition. The flies are held in the pots for seven to ten days until they lay their eggs. The average number of eggs laid per fly depends on the species and nutrition but usually averages between 30 and 38 eggs (range 1-100+) per gravid female. Therefore, the expected number of eggs in a 125-ml pot loaded with 50 engorged females will be approximately 1500-1900 and 4500-5700 in a 500-ml pot loaded with 150 females. Most species lay fewer eggs under crowded conditions. For example, *P. papatasi* oviposit optimally in pots with 100-120 females but deposit fewer eggs if female numbers are increased to 200 or more (Figure 12).

#### Low fertility

Occasionally, low fertility is observed in sand fly colonies where females oviposit but only a small percentage of the eggs hatch. Reasons for this may have to do with the length of time that the females and males are held together (before, during or after feeding) and whether they prefer to copulate in large spaces (eurygamy) or small spaces (stenogamy). With colonies that are initially difficult to colonize because of low fertility, experimenting with different size cages and periods of female-male exposure (before, during and after engorgement) may prove fruitful. From work done with *Phlebotomus ariasi* Tonnoir, 1921, it was observed that female flies that fed in a mosquito net made with fine gauze (68 × 68 × 185 cm; ∼28 × 28 × 72 in) and that were left with males for 24 hrs after engorgement, produced eggs with a high rate of hatching [28]. After several experiments using large- and small-size fabric-net cages, these workers were able to optimize the cage size at 45 × 45 × 45 cm (∼18 × 18 × 18 in), and by leaving the males in the cage with the females for 24 hrs after engorgement, two robust colonies of this species were established.

### Rearing immature stages

#### Eggs

**Figure 13:**
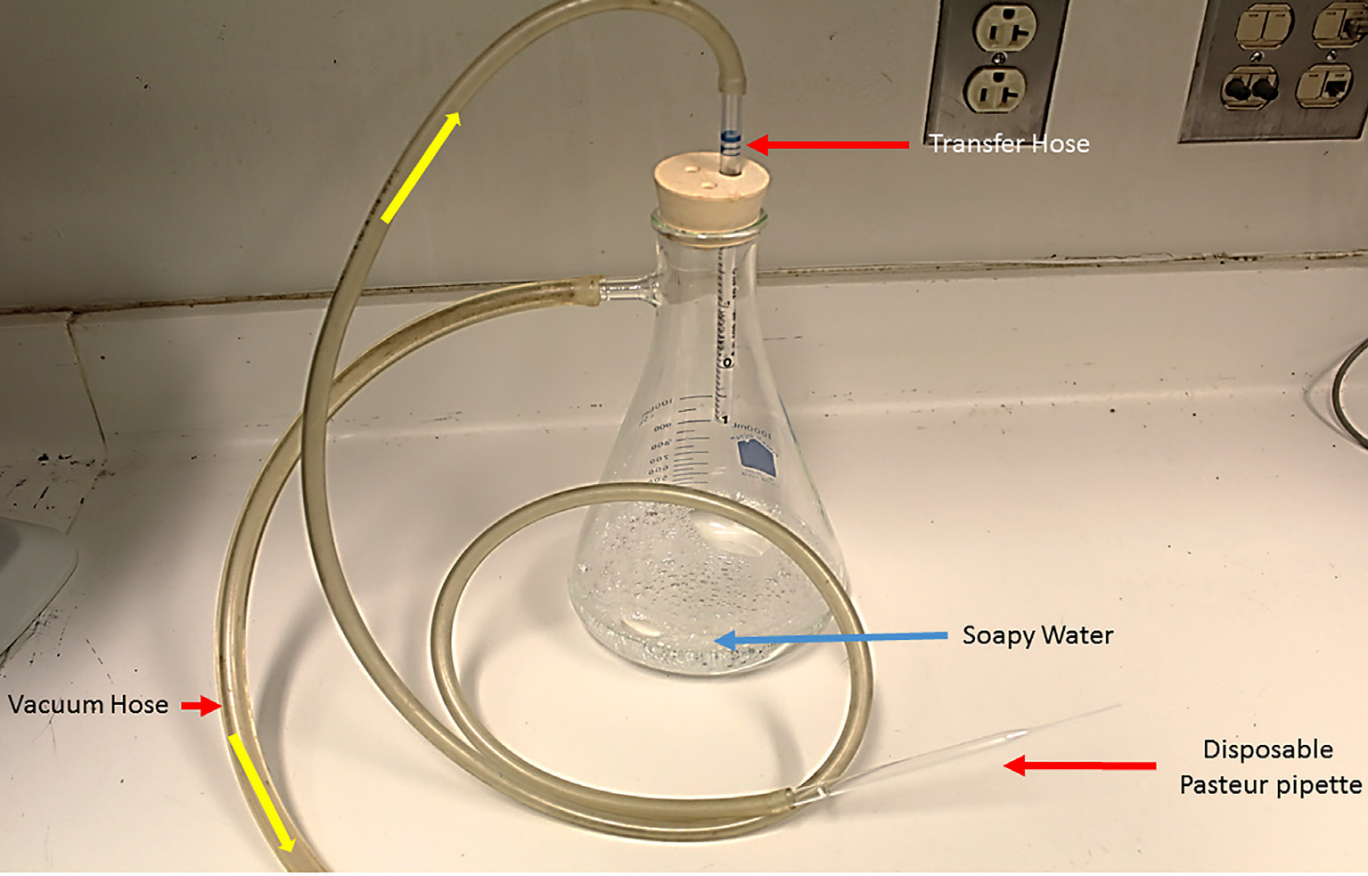
Vacuum-powered pipette aspirator for removing dead adults and mites from ovipots. Arrows indicate the direction of air flow. (Photo by T. Rowland).

Following oviposition, the surviving flies are either removed *via* forceps or vacuum aspirator, or released back into the colony mating cage to start another gonotrophic cycle. For large colonies with several hundred to a thousand or more blood-fed females per feed, the number of ovipots per generation may be as many as 10 or more. To save time, live and dead flies can be removed quickly from the pots with an inexpensive, custom-made vacuum-powered pipette aspirator set at low pressure, without removing the eggs (Figure 13). At the same time, any mites observed in the pot can also be removed. Removal of the flies from the pots is critical to prevent excessive mould growth that might entrap the first instars, to reduce phorid mite infestations, and to prevent the larvae from eating the adult fly carcasses and becoming infected with gregarines or other vertically transmitted pathogens. With the adults removed, the eggs are washed with a 1% sodium hypochlorite solution to remove gregarine oocysts adhered to the exterior surfaces of the eggs (see Appendix D for egg washing procedure). The washed eggs are then rinsed with clean tap water and placed back into oviposition/rearing pots. The 500-ml oviposition pots become rearing pots with the addition of solid plastic, vented lids (Figure 10c), which keep the pots aerated and prevent condensation build-up on the interior wall of the pot, at the same time maintaining the humidity level in the pot. Rearing pots with unhatched eggs are stored in plastic boxes (Figure 8a) or, with the solid, vented lids, they can be stacked in open trays (Figure 8b) and placed inside an environmental cabinet at 25°C and 80% RH. The pots are then monitored at least three times per week for hatching and for the presence of mites.

#### Larvae

Equally as important as successful feeding of the adult females is successful feeding of the larvae. R. Killick-Kendrick stated that “Among the major problems in rearing phlebotomine sand flies is excessive larval mortality caused by fungal growth, improper diet or moisture, disease and other factors.” [24] These problems are especially acute during the first stadium when the tiny first instars (Figure 7c) become easily entrapped in fungal mycelia or in moisture condensation. When the eggs hatch, a small amount of larva food (a composted 1:1 mixture of rabbit chow and rabbit faeces; see Appendix C) is sprinkled on the surface of the plaster lining the bottom of the pot. A small container with a perforated lid or one covered with a fine gauze top secured with an open plastic cap (with a hole cut in the middle) works well for this purpose. The fine particles are easy for the first instars to manage. Occasionally, high first-instar mortality occurs in spite of best efforts because the larvae, upon eclosion are weak and slow, and find it difficult to reach food particles. One of the authors (MK-K) observed while working with a colony of *Lu. youngi* Murillo & Zeledon, 1985 that when all the eggs in a pot were gently brushed toward the centre of the ovipot and then very fine food was sprinkled around the eggs, the first instars moved toward the wall of the pot as if seeking a hiding place for protection. However, when the eggs were encircled with frass (spent food) from another pot in which larvae had fed, and then fine food was sprinkled over the frass, the first instars fed very well, underscoring the fact that breeding sand flies is more than just following “recipes”; one must try to mimic what happens in nature. In other words, “think and feel like a sand fly.” (MK-K). Larva pots are checked at least three times per week and the food is replenished according to the number of larvae and their size. Overfeeding leads to fungal growth and underfeeding to cannibalism and unequal development. As the food is digested and passes through the larvae, it becomes finely granulated frass. The amount of food to be given is gauged by the proportion of frass in the pot. In a healthy pot, the larvae will turn all the food to frass in a couple of days. As the larvae develop from first to fourth instars (usually about 20-25 days [58]), they require progressively more food until the late fourth instar (Figure 7f), when feeding stops and pupation begins (Figure 7g). At each feeding, the pot is shaken gently to stir up the food and break up fungal growth. An abnormally long generation time may indicate that the colony is suffering from pathogens, unfavourable rearing conditions or the larvae may be entering diapause, any of which is bad for laboratory use.

##### Moisture control in rearing pots

Moisture in each pot is monitored regularly, visually and by feeling the bottom of the pot. The plaster in the rearing pots can be re-saturated as needed but one must be careful not to over-saturate, otherwise fungus will develop. Oversaturation may also cause the larvae to unnaturally climb the sides of the containers in search of dryer conditions. This can be remedied by placing the freshly saturated pots on absorbent paper or cotton towels for a few minutes to absorb excess moisture. Two different methods commonly used to maintain adequate moisture in larva pots are described below. Both methods work well. The method employed may depend on the type of pots used, colony size, storage capacity, local resources, or user preference.

###### With sand

This method works well with larva pots fitted with open-screen lids (Figure 10a). Pots with larvae are stored in plastic boxes. To maintain adequate humidity, the bottoms of the plastic storage boxes are filled with a ∼1 cm-deep layer of fine sand dampened with distilled or bottled water. (Figure 10a.) For maintenance of colonies, sand is better than filter paper because of higher water-retention capacity. Moreover, the sand can be recycled easily as it is washed and sterilized before use. Shaking or stirring the sand layer at least weekly prevents fungal growth on the bottom of the container. The easiest sand to obtain is the type used in construction that comes in large bags; it is fine and pre-cleaned. River sand can be used if it is washed several times to remove impurities. The same is true with sea sand, which must be washed thoroughly to eliminate salt. Another possibility is to use small clay beads used for growing plants; they retain the moisture and are good for avoiding overwatering. The sand and clay beads should be washed and sterilized in an oven at 100°C for 1 hr, after each generation before reusing. Storage should be in a clean container with closed lid and in a dry and cool place.

###### Without sand

This method works best with larva pots fitted with vented solid plastic lids (Figure 10c). Pots with larvae are stored in large photographic trays inside an environmental cabinet and can be stacked on top of each other (Figure 8b). This method is especially useful for large colonies where storage space is limited. The vented solid plastic lids reduce evaporation from the water-saturated plaster in the bottom of the pot, which keeps the larva food moist. The vent in the centre of the lid, which is covered with larva proof netting, allows for air circulation and prevents water condensation on the sides of the pot. The bottom of each pot is checked when the larvae are fed to make sure it is moist and, if necessary, the pot is placed in a tray with water for about five minutes to re-saturate the plaster. After re-saturation, the pot is placed on an absorbent cotton towel to take up excess water and the bottom of the pot is wiped clean.

##### Larva food

Although most sand fly species that have been colonized successfully thrive on a composted 1:1 mixture of rabbit faeces and rabbit chow [39,47,48,57,65], with some species that are difficult to colonize, it may be necessary to replace the rabbit faeces with faeces of other wild or domestic animals taken from the places where the flies live. In the early stages of colony initiation, it is good to prepare batches of food with different ingredients, based on field observations and one's own imagination, and see which recipe is most acceptable to the larvae. At the Kala-azar Medical Research Centre, Muzaffarpur, Bihar, India, a colony of *P. Argentipes* was initiated and established from wild stock collected in nearby villages [57]. Initially, larva food consisting of a composted 1:1 mixture of commercial rabbit chow and rabbit faeces was provided by the Division of Entomology, WRAIR, until suitable food could be prepared at the Kala Azar Medical Research Centre (KAMRC) from locally available ingredients. A variety of natural ingredients such as rabbit faeces, cow manure and goat faeces, all readily available at the collection sites, were composted 1:1 with locally purchased rabbit chow to make larva food. Food made with locally collected rabbit faeces and locally purchased rabbit chow did not work well, owing perhaps to excess moisture and urine content of the faeces. Surprisingly, composted cow manure was not well accepted by the larvae. Finally, dried goat faeces, aerobically composted 1:1 with locally purchased rabbit chow, made excellent larva food and is now used to maintain the colony [57]. The larvae of cave dwelling sand flies may prefer food made with faeces of animals that frequent the caves for water or salt, such as antelope and elephants, or rodents and bats that actually live in the caves. Killick-Kendrick and Killick-Kendrick [25] found that adding protein, such as *Daphnia* or dried leaves of plants that occur in places where the larvae have been found may make the food more acceptable, as with *Lu. youngi* in Brazil. These authors also reported that free-living nematodes, accidentally introduced into larva food were found to be beneficial [25] (see paragraph 5.5). Because the aerobic composting process [39,48,49,59,67] takes two weeks to one month, the food must be prepared well in advance of initiating a colony so as to be available to feed the developing larvae. See Appendix C for larva food preparation procedures.

#### Pupae

Toward the end of the fourth stadium, the larva stops feeding, and depending on species and maintenance conditions, may migrate to the drier portion of the substrate; it then evacuates its gut and becomes opaque white and swollen at the anterior end (pre-pupa). The pre-pupa attaches to the substrate surface, the side of the pot or the underside of the lid and pupates (Figure 7g). Pupae are sessile and non-feeding, but they are active and respond to stimulation by wagging. As the pupa matures, it turns dark in most species. Caution must be taken when removing the lid of the containers so as not to injure pupae that are attached to the underside of the lid.

#### Dealing with diapause

Many species, especially in temperate climes and in arid regions, produce a proportion of eggs or fourth instar larvae that will enter a facultative diapause triggered by various environmental cues such as decreasing photoperiod or temperature [40]. As mentioned in paragraph 2.2 above, *Lu. diabolica* in south-central Texas, USA begin laying diapause eggs as early as 1 September that do not hatch until April or May of the following year [40]. Volf *et al.* [59] observed a very long larval diapause and generation time of almost one year in the Mediterranean species *Phlebotomus simicii* Artimiev 1974, with adults emerging under laboratory conditions 197-335 days after the eggs were laid. In such cases, it is best to collect flies for colony stock at the beginning of the season, during the first population peak, when they are unlikely to diapause, thus avoiding the harvesting of diapausing eggs or larvae. Other species, such as *P. ariasi,* may undergo several months of obligatory diapause, making laboratory colonization extremely difficult. In work done with *P. ariasi,* the rearing temperature was lowered incrementally, 4−5°C at a time from 24°C to 10°C, over a minimum period of three weeks, after which the larvae were kept at 10°C for a minimum of three months, in total darkness. Pots were checked weekly for fungus and a little food was added if needed. After three months, the incubator temperature was raised incrementally 4−5°C at a time over three weeks, and the larvae pupated. Some pots with larvae in diapause were kept for even longer periods, up to 1 year, with the same results (MK-K, work unpublished) [55].

### Adult Emergence

**Figure 14:**
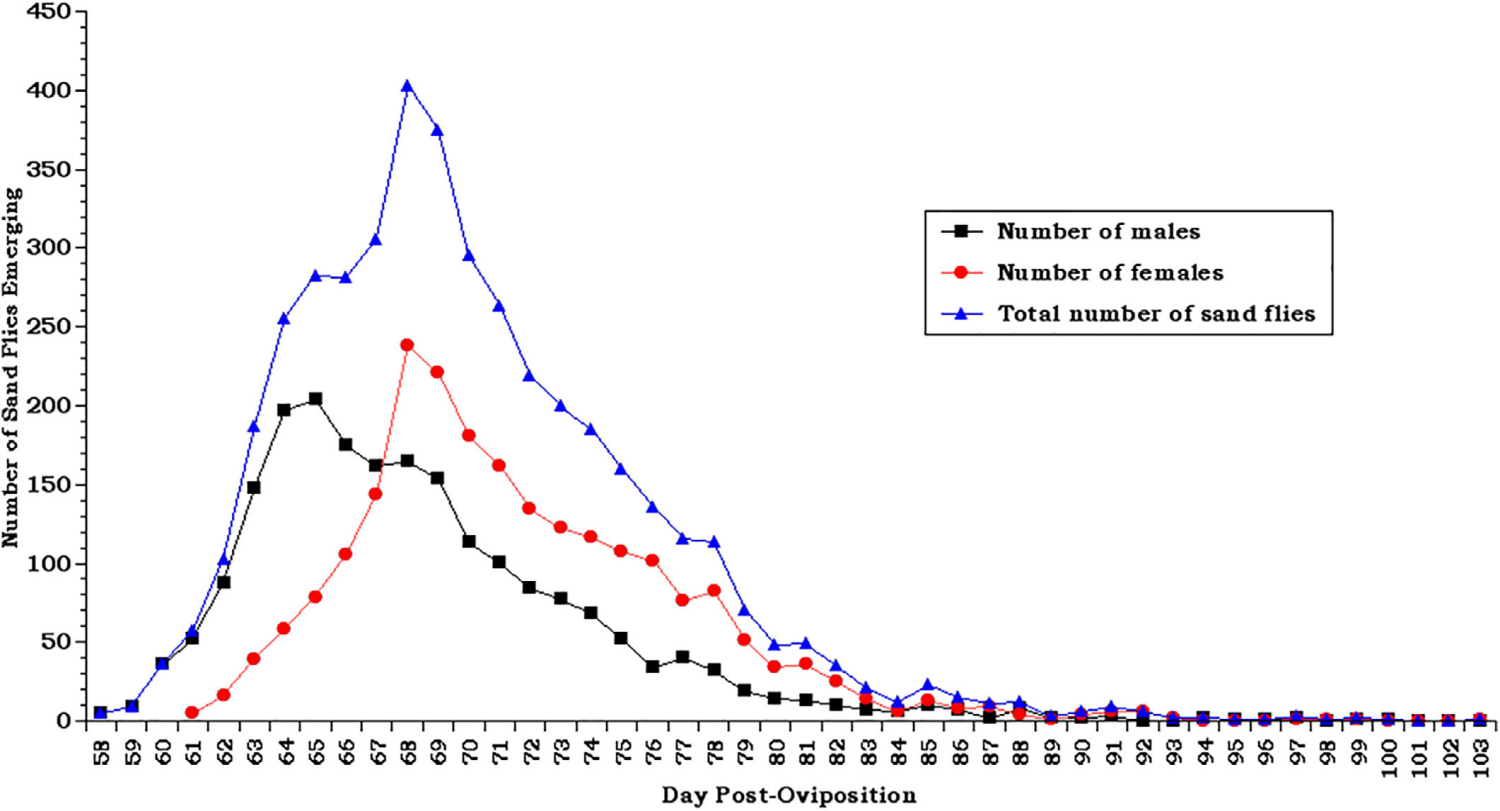
Adult emergence profile of *Lu. verrucarum* derived from isoline rearing of 100 broods at 24°C and 70% RH.

Adults emerge roughly 7-11 days following pupation. Males usually precede females by one or two days (Figure 14). Upon emergence, the genital armatures of males are upside-down and must rotate 180 degrees before the male are sexually mature, which may take on average as little as 12 hrs in *Sergentomyia schwetzi* (Adler, Theodor and Parrot) to as long as 33 hrs in *Phlebotomus sergenti* Parrot, 1917 [61], coinciding with the emergence of females that are sexually mature upon emergence. Depending on the species, adults in a rearing pot will emerge over a period of a few days to as long as two months. Some species, such as *Lu. longipalpis* and *P. argentipes* exhibit synchronous emergence, with all adults emerging over two or three days, whereas species like *P. papatasi* and *P. duboscqi* exhibit semi-asynchronous emergence over a period of several weeks to two months or more. Emerged flies are released from rearing pots two or three times weekly into mating/feeding cages and the cycle is repeated. To prevent flies from escaping into the insectary during the release, a large elastic band is slipped over the cage sleeve so that it fits snuggly over the pot and the releaser's arm as it is introduced into the cage. The pot lids are removed inside the cage and the pots shaken to encourage the adults to fly out of the pot and into the cage. After release, the lids are replaced and tightened inside the cage and any flies resting on the lid or the pot are brushed away before removing the pot from the cage.

#### Managing escapees

In the process of handling large numbers of adult sand flies, a few inevitably will escape into the insectary. Sand flies disappear quickly even in an insectary where the walls are painted white; success of capturing or killing them depends largely on response time. When sand flies escape they usually land high on vertical surfaces and tend to congregate in corners. They can be recaptured with a mouth aspirator or with CDC-type or other type light traps fitted with incandescent or ultraviolet lights. Place the trap near the area where the sand flies were last spotted, then turn out all the lights and leave the area for 12 hours. Successful recapture can be enhanced with CO_2_-bait, either from a compressed-gas cylinder or from dry ice placed in a thermos. Do not return live, recaptured escapees to the colony. Sticky traps work well when placed on surfaces where flies are likely to land such as in corners, at the tops of walls where they join with the ceiling, and under counters. Manual fly swatters and a variety of electric fly swatters (such as Executioner^®^ purchased from Amazon.com) can be used effectively to kill escapees. Ethanol (70%) sprayed in a fine stream directly at escaped flies is very effective. Precautions must be taken to ensure there are no electrical outlets or open flames in the area being sprayed.

## Parasites and pathogens

Without going into great detail, suffice it to say that natural parasite infections in wild-caught sand flies are not uncommon. Young and Lewis [66] and Warburg *et al.* [62] summarized the published reports (excluding mites) that have been observed in phlebotomines. Adult sand flies have been found naturally infected with a variety of protozoans, nematodes, cestodes, fungi, bacteria and viruses. Lawyer [38], working with *Lu. diabolica* in south-central Texas, USA, found natural parasite infections in (or on) 341 female sand flies during two summers of field collections, including ciliated protozoans, flagellated protozoans, microsporidians, gregarines, nematodes, mites, fungi, and bacteria. While the significance of infections with several of these organisms is uncertain, some are known to be deleterious to colonies and some may even be beneficial.

### Gregarines

**Figure 15:**
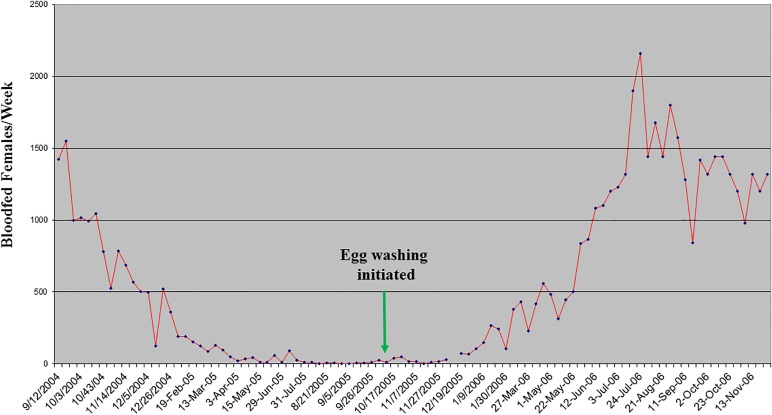
Graph showing the crash and near extinction of a working laboratory colony of *P. papatasi* caused by infection with aseptate gregarines and subsequent recovery after commencement of egg-washing and treatment procedures.

**Figure 16:**
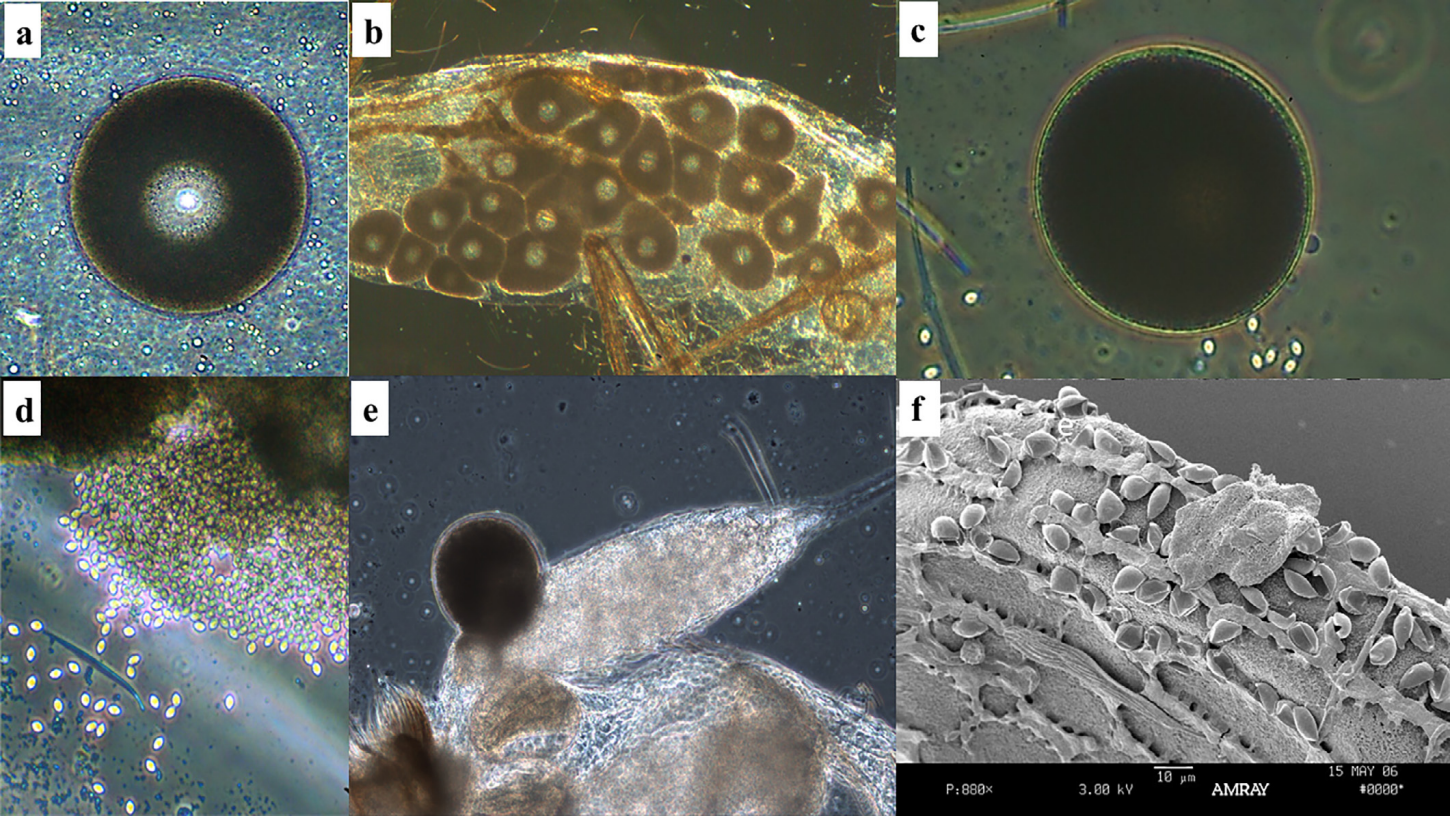
Life cycle of aseptate gregarines in *P. papatasi*: a. Trophozoite (gamont) in the hemocoel of a female sand fly; b. Heavy infection with gregarine trophozoites in the haemocoel of male *P. papatasi*; c. Gametocyst (Note the absence of a nucleus); d. Oocysts spilling from the lumen of an accessory gland; e. Gametocyst attached to the accessory gland of the female sand fly; f. Oocysts adhered to the exterior surface of a sand fly egg. (Photos a–e by P. Lawyer; photo f. by E. Asafo-adjei).

**Figure 17:**
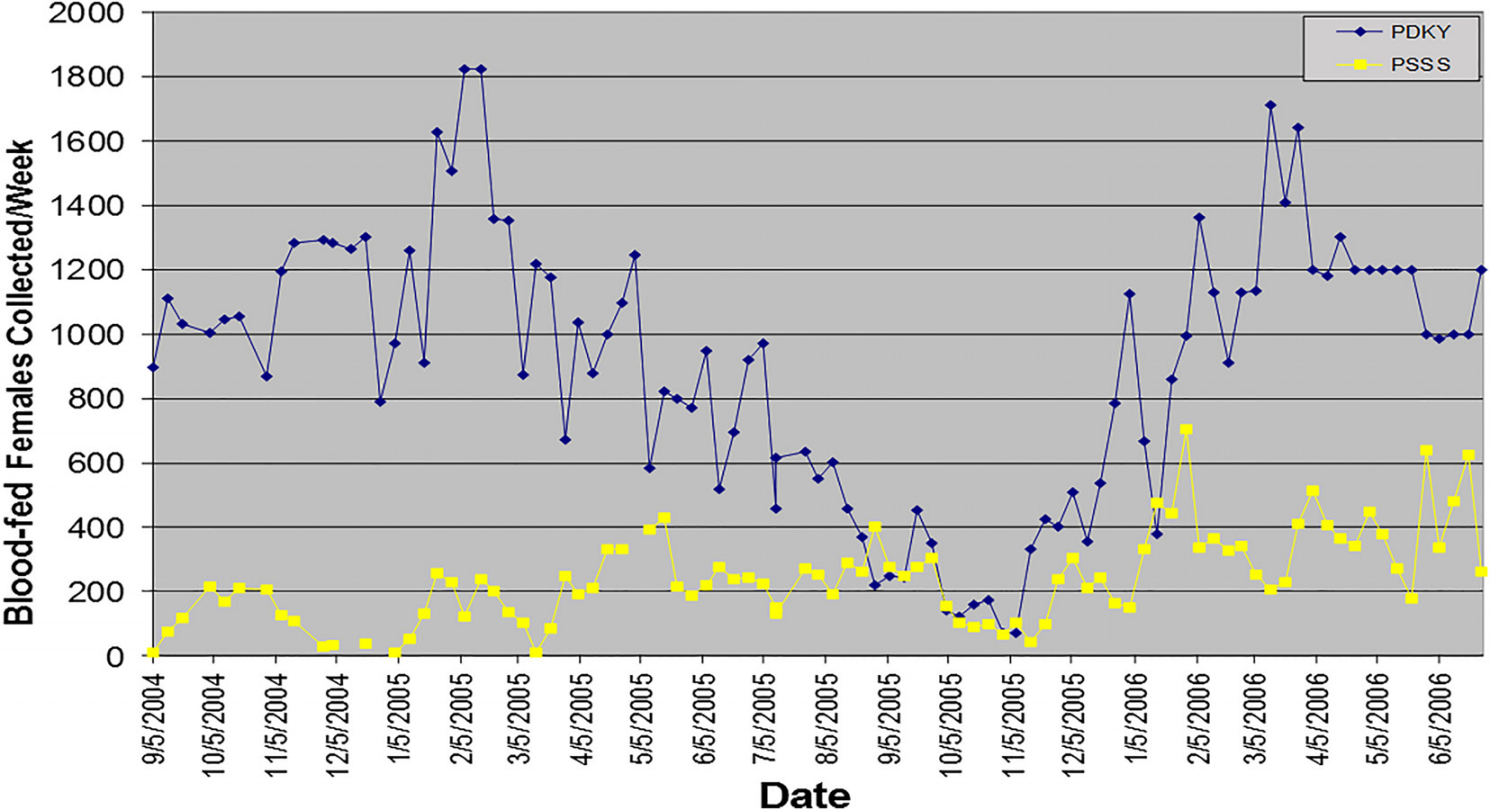
Graph showing recovery of two sand fly colonies following washing of eggs with 1% sodium hypochlorite solution. The *P. duboscqi* colony (PDKY, blue line) responded quickly to the treatment compared to the more gradual response of the *P. sergenti* colony (PSSS, yellow line) infected with *Psy sergenti*.

**Figure 18:**
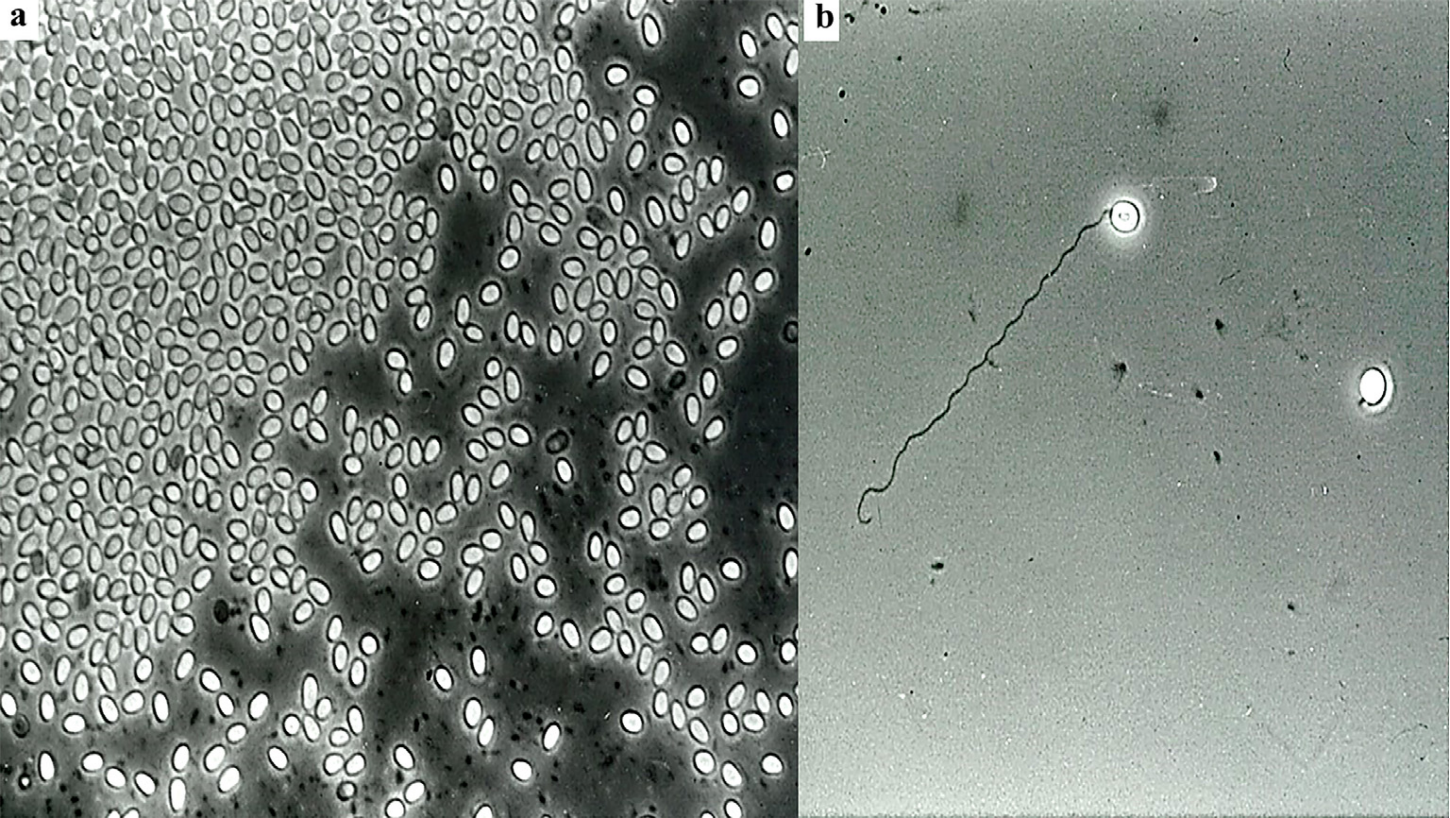
a. Microsporidian spores in the haemocoel of a *Lu. diabolica* female collected at Garner State Park, Uvalde County, Texas (approximate magnification × 2100; photo by P. Lawyer); b. Microsporidian spore germinated *in vitro* by addition of 0.2 M KCL (pH 9) to dissecting medium (insect Ringer's solution, pH 7.2) (approximate magnification × 2640; photo by P. Lawyer) [38].

Gregarines are quite common in laboratory sand fly colonies. Dougherty and Ward [14] reported that in *Lu. longipalpis* colonies, the level of parasitemia was 70-90%. Similar levels of gregarine parasitemia have been seen in colonies in other laboratories. These parasites occur naturally in wild sand fly populations and appear to enjoy a commensal relationship with the sand fly without significant negative effects. However, under intensive mass rearing, high parasitemia can develop, leading to reduced longevity and fecundity and severe decline of the colony population. For example, over a one-year period from about September 2004 through November 2005, the principal working colonies at the WRAIR experienced a phenomenon referred to as colony crash, with populations dropping to such low levels that sand flies needed in critical research were unavailable and the survival of the colonies was in jeopardy (Figure 15). Upon microscopic examination, all of the working colonies were found to be heavily infected with aseptate gregarines (Figure 16a). Aseptate gregarines parasitizing sand flies belong to the genus *Psychodiella* [60]. Currently, five *Psychodiella* species are described; the review on their taxonomy, life cycles, host specificity and pathogenicity is given by Lantova and Volf [37]. The exact mechanisms of pathology caused by the gregarines is unknown but it is not difficult to imagine that an infection such as that shown in Figure 16b in the haemocoel of a male *P. papatasi* could have negative effects. Briefly, two mature trophozoites come together and undergo pseudoconjugation within the sand fly to produce a gametocyst (Figure 16c). They then undergo nuclear division within the gametocyst to form numerous gametes, which in turn, conjugate to form zygotes. Each fully grown zygote secretes a tightly fitting shell and becomes an oocyst (16d). Some gametocysts become attached to the accessory glands of the female sand fly and, as internal pressure develops within the gametocyst, its wall ruptures, liberating mature oocysts into the lumen of the accessory gland (Figure 16e). During oviposition, the surface of the sand fly egg is coated with the infected accessory fluid. When the larvae emerge they eat the egg cases with the attached gregarine oocysts (Figure 16f), as well as parasitized dead adults. (See also [36]) Gregarine control begins with removing the dead adults from the oviposition pot either with forceps or with a vacuum aspirator, then washing the eggs with formol or sodium hypochlorite to remove the oocysts from the surface of the egg shell. Dougherty and Ward [14] were able to reduce parasitemia levels of *Ascogregarina chagasi* Adler & Mayrink, 1961 in *Lu. longipalpis* by 86.3% by washing eggs with 0.1% formol. Similarly, PGL (work unpublished) washed the eggs of the WRAIR colonies with 1% sodium hypochlorite for one minute, twice weekly for six months and all but eliminated the parasites from the colonies (Figure 17; see Appendix D for egg washing procedures). Interestingly, the *P. sergenti* colony was infected with a different gregarine, *Psychodiella sergenti* Lantova, Ghosh, Svobodova, Braig, Rowton, Weina, Volf & Votypka 2010 [36,37], which did not succumb as quickly to washing with 1.0% sodium hypochlorite (Figure 18). Jancarova, Hlavacova, Votypka and Volf [21], were able to eliminate *Psy.* *sergenti* from a *Phlebotomus sergenti* colony by raising the colony rearing temperature from 27°C to 32°C. The increased rearing temperature affected the larval developmental times and size of *P. sergenti* adults but had no effect on the susceptibility of *P. sergenti* to *Leishmania tropica* Wright, 1903. This temperature manipulation may work for other gregarine-infected sand fly species as well.

### Microsporidians

Microsporidian infections have been reported in several Old- and New World sand fly species and some can cause high mortalities in their hosts under laboratory conditions (Figure 18) [9]. Fortunately, their occurrence in laboratory colonies is rare.

### Mites

**Figure 19:**
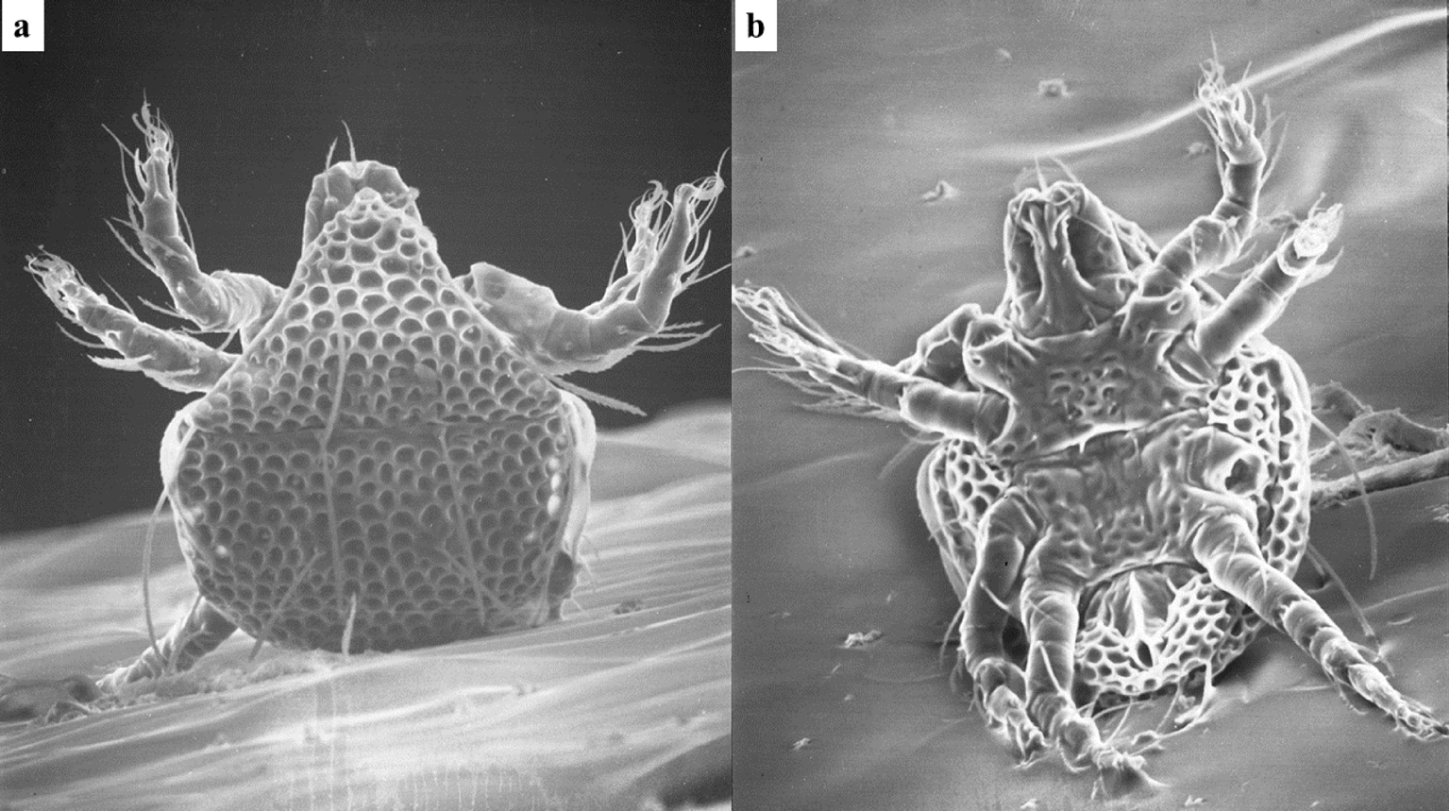
Scanning electron micrographs of a phorid mite (*Eustigmaeus* sp.) found on the abdomen of a female *Lu. diabolica* collected at Garner State Park, Uvalde County, Texas: a. Dorsal aspect, magn. x 1704); b. Ventral aspect, magn. × 1700). (Photos by P. Lawyer).

Mites are ubiquitous and almost impossible to avoid (Figure 19). Lewis and Macfarlane [41] reported that 14 families (particularly Stigmaeidae), and at least 16 genera and 21 species have been found on 39 species of sand fly. According to these authors, mites may be phoretic, parasitic or both. Attachment scars left by the mites may be harmful to the host. Some mites are entomophagic and in large numbers can quickly overwhelm a pot of larvae. For best results, mite control must begin as soon as possible after oviposition with the meticulous removal of sand fly carcasses (including legs and wings lost during oviposition) from the ovipot with ultra-fine forceps or vacuum pipette aspirator (Figure 13). At the same time, carefully examine the interior surfaces of each pot and remove or kill any mites or mite eggs that are visible. While adult mites and older nymphs are usually clearly visible with the naked eye, a microscope will be needed to see the mite larvae and eggs. After the sand fly eggs are washed for gregarine control, always return them to a cleaned pot. Ovipots should be checked daily for mites until the eggs hatch and at least weekly thereafter. Once the sand fly adults have emerged, place the used pots in a −20°C or colder freezer for at least 24 hours. Wash the ovipots in hot water (no detergent) and scrub the surface of the plaster layer in the bottom of the pot clean. Store the pots upside-down in clean trays until use. Some labs change the plaster in the pots after each generation, but others have found that, with proper cleaning and scraping, this is not necessary and, in fact, females lay their eggs more readily in pots with used plaster. Some labs keep the pots at 28°C to discourage mite infestation. It is also extremely important to wash and sanitize with 70% ethanol all surfaces on which the rearing pots are to be placed including countertops, plastic storage boxes and trays, and incubator shelves to prevent re-infestation. Mite sprays have been tried with limited success but their prolonged use has not been evaluated. In one NIH insectary, eggs are treated twice monthly for 10 minutes with a mite spray, developed for use with *Drosophila* colonies [http://flystuff.com/drosophila-products/mite-control/], before the usual washing and treatment for gregarine control. The spray is harmless to the eggs and they hatch without any problems. However, the prolonged use of the mite spray has not been evaluated (Claudio Meneses, personal communication).

### Fungal and bacterial infections

Control of fungal growth/moulds in larva pots is best accomplished by proper aerobic composting of the larva food ingredients to produce white, cushiony moulds that the larvae readily eat, and by properly measured introduction of the food. When hatching begins, sprinkle only a very small amount of finely ground food on the plaster surface in the bottom of the pot in the areas where the first instars appear and monitor the pot daily to insure that the food is being eaten and that harmful mould-growth does not occur. When larval density in a pot is low, mould will often grow out of control and entrap the larvae but when density is high the larvae, feeding in mass, will usually keep the mould under control. Screen tops on cages or on oviposition pots where sugar-soaked cotton balls are placed must be cleaned daily and changed frequently to prevent harmful mould growth. It should be kept in mind that in nature, soil with the existing fungal and bacterial fauna, is an ideal place for interrelationships between living organisms. What is offered to sand fly larvae in the laboratory probably does not adequately replicate what occurs in nature. Most bacteria eaten by larvae are defecated with the midgut content before pupation, however, some can passage transstadially to adults, which acquire other bacteria from the environment and sugar meals [59]. The significance of infection by these organisms is uncertain. In some cases, infection with certain bacteria may cause premature death of the fly while other bacteria may actually be beneficial to the fly [52]. In laboratory colonies, bacterial infections in adult sand flies can be controlled with penicillin/streptomycin added to the sugar meals. However, if the flies are to be used for *Leishmania* infections and transmission, they should not be fed sugars treated with streptomycin, tetracycline, or other antibiotics that interfere with leishmanial metacyclogenesis. Recently, it was demonstrated that midgut microbiota play an essential role in sand fly vector competence for *Leishmania* [23].

### Nematodes

Nematodes of several families have been recovered from the body cavity of phlebotomine sand flies [30,53]. Poinar, Ferro Morales and Tesh [54] conducted laboratory studies indicating that heavy infections of a tylenchid nematode (*Anandranema phlebotophaga* Poinar, Ferro, Morales, & Tesh, 1993), found in *Lu. longipalpis* can cause sterility in female flies or reduce egg production. These workers conducted further laboratory trials using *Heterorhabditis* sp. (Heterorhabditida: Rhabditida) and *Steinernema carpocapsae* Weiser, 1955 (Steinemematidae: Rhabditida) against larvae of the sand fly*P. papatasi* and showed that infection with both nematodes could occur with varying degrees of mortality. On the other hand, in the colonization of *Lu. youngi*, from Venezuela, free living nematodes (*Ceonorhabditis sp* and *Aphelenchoides bicaudatus* Hunt, 1993), presumably introduced into the larva food with the addition of ground coffee leaves, were found to be very useful for the control of bacteria and fungus. The nematodes are very difficult to notice in a breeding pot because they hide from the light. The best way to see them is to put a small amount of frass taken from the breeding pot in a Petri dish with some distilled water and examine it under a stereoscope with a light projecting from below the stage. As *A. bicaudatus* feeds on fungi and *Coenorhabditis* on bacteria, the pots were kept clean and the frass was like friable compost. The nematodes did not harm the larvae and may even have served as a protein source [25].

## Good Housekeeping and Cleanliness

Good housekeeping and cleanliness in the insectary cannot be over emphasized. All surfaces on which cages or pots are placed must be kept neat and clean. Insectary staff should wash bench tops daily with soap and water and sanitize them with 70% ethanol. Likewise, floors should be swept and mopped daily. No chemicals should be used on the floors, walls or bench tops, without first being tested for toxicity to the sand flies. Also, sand fly colonies should not be housed in insectaries where mosquitoes are colonized because the high humidity and heat will promote mould and fungus. To prevent contamination, all handling equipment, incubators and rooms used for colonization should be kept clean through sterilization of material if possible, thoroughly washing or changing the plaster in the rearing pots after each generation, washing lids, trays, nets and cages and all transfer apparatus. Incubators should be wiped down regularly with 70% ethanol (not isopropanol). Technicians should be instructed not to use hand lotions while working with the sand flies.

## Disclaimer

This material has been reviewed by the Walter Reed Army Institute of Research. There is no objection to its presentation and/or publication. The opinions or assertions contained herein are the private views of the authors, and are not to be construed as official, or as reflecting true views of the United States Department of the Army or the Department of Defense. Research was conducted in compliance with the Animal Welfare Act and other U.S. federal statutes and regulations relating to animals and experiments involving animals, and adheres to principles stated in the *Guide for the Care and Use of Laboratory Animals*, NRC Publication, 2011 edition.

## Figures and Tables

**Figure C:**
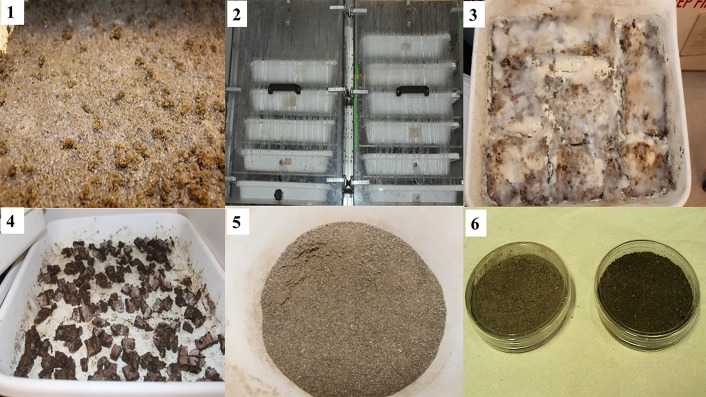
1 – The mixed ingredients (rabbit chow and rabbit faeces) are saturated with water and spread out evenly in a shallow tray; 2 – Trays loaded with the food ingredients are placed on racks in a custom-made composting cabinet and the cabinet door is sealed shut; 3 – After two weeks of composting, the door of the cabinet is removed and the finished larval food is air dried for two or three days; 4 – Composted and dried larva food ready for grinding; 5 – Ground larva food ready for use or storage; 6 – light-coloured food on left** **=** **not good; dark-coloured food on right** **=** **good. (Photos 1-5 by Tobin Rowland; photo 6 by Claudio Meneses).

**Figure D:**
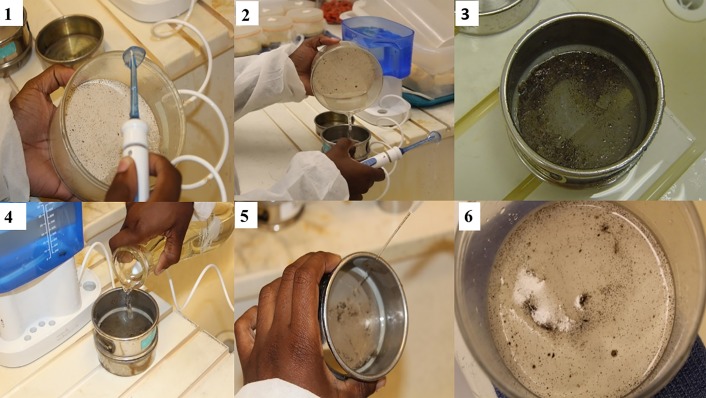
1 – Sand fly eggs are dislodged from the plaster surface in the bottom of an ovipot using a Waterpik^®^ (oral irrigator) with fan-spray attachment; 2 – The egg/water suspension is flushed into the appropriate colour-coded sieve; 3 – Eggs in a sieve that have been flushed from an ovipot; 4 – The sieve containing eggs is nested in a soaking pan filled with 1% sodium hypochlorite; 5 – After soaking, the eggs are flushed to one side of the sieve with a squirt bottle; 6 – The eggs are flushed from the sieve and distributed evenly over the plaster surface in the bottom of a clean ovipot. (Photos by T. Rowland).

## Appendix C Preparing larva food

### Materials

- rabbit faeces;
- rabbit chow;
- plastic 19-liter tub (5 gal.);
- water;
- cat litter scoop;
- 15.2-cm (6 in.) wide plastic spatula;
- polycarbonate larva food composting cabinet;
- 46 × 66 × 8 cm (18 × 26 × 3 in) photographic developing trays;
- gloves;
- lab coat;
- face mask.

### Procedures

It is essential that larva food preparation begins well in advance of anticipated need, otherwise larvae will starve and the colony will decline or die. Various larval diets and methods of larva food preparation are described in the literature (Young *et al.*, 1981; Modi & Tesh 1983; Volf & Volfova 2011). The system described here enables preparation of quality sand fly food in sufficient quantity to keep pace with the demands of mass rearing. The ingredients, fresh rabbit faeces from a local animal facility and rabbit chow (Pro-lab Rabbit Chow) are mixed 1:1 in a large plastic tub (45 × 30 × 22.5 cm; 18 × 12 × 9 in) with warm/tepid water and stirred continuously until the water is absorbed into the mixture (approximately four liters (one gallon) of each ingredient in nine liters of warm water). More water can be added as needed to make sure the mixture is well saturated but not muddy (no standing water). When the mixture is thoroughly saturated, it is scooped into a shallow plastic photographic developing tray (46 × 66 × 8 cm; 18 × 26 × 3 in) and spread out evenly to a depth of approximately 2 cm (< 1 in) (Figure C-1). No portion of the tray bottom surface should be visible. Next, the trays are placed on racks in a custom-made composting cabinet (Figure C-2), a pan of warm water is placed on the very bottom of the cabinet and the cabinet door is sealed shut with upper and lower vent covers ¾ open. The vent openings permit free flow of gases out of the cabinet and promote aerobic composting. If properly composted, the final product will smell pleasant like humus or potting soil and will be well accepted by the larvae. If composting is anaerobic, putrefaction will occur and the food will have a foul odour and will not be good for the larvae. Frequent removal of the cabinet doors to further ventilate the food will enhance aerobic composting. After one week of composting, remove the cabinet door and, one-by-one, remove each of the trays. Using a large scoop or spatula, flip the larva food over and mix it thoroughly. Spray the surface with water and replace the trays in the cabinet in reverse order of the way they were originally placed in the cabinet, *i.e.* bottom tray to the top shelf and so on. Replace and seal the cabinet door and let the composting continue for one more week with the two vent covers ¾ open. After two weeks, remove the cabinet door and allow the finished larval food to air dry for two or three days (Figure C-3). When the food is completely dry (Figure C-4), scrape it from the composting trays and grind it in a grinding mill and store it in 4-liter plastic buckets, or other sealable containers, in a −30°C freezer (Figure C-5).

## Appendix D Egg washing procedure for gregarine control

### Materials needed

- disposable gloves;
- lab coat or apron;
- 0.0875-cm mesh soil test sieves (0.0035-in mesh); one for each colony, colour-coded by colony;
- 7.5-cm (3 in) diameter brass capture pan;
- coloured tape for colour coding sieves by colony;
- 6.15% sodium hypochlorite solution (Clorox^®^) or other locally available commercial bleach solution;
- 500-ml squirt bottle;
- glass Pasteur pipette;
- 1000-ml beaker;
- 500-ml graduated cylinder;
- distilled water or deionized water;
- minute timer;
- Lab towels;
- Waterpik^®^ oral irrigation apparatus; if a Waterpik is not available, a squirt bottle can be used.

### Procedure

Make up a 1% hypochlorite solution. If using Clorox^®^ (The Clorox Company, Oakland, CA, USA), which is 6.15% sodium hypochlorite, measure 162.6 ml of the stock solution with 837.4 ml in a 1000-ml Erlenmeyer flask. Fresh solution should be made on the day of each egg washing. Fill the Waterpik reservoir with distilled or deionized water. Turn the Waterpik on and with the fan spray attachment, flush the inside of the ovipot to dislodge the eggs (Figure D-1). Next, flush the egg/water suspension into the appropriate colour-coded sieve (Figure D-2). Place/nest the sieve that now contains only the eggs into the brass soaking pan. Gently pour 1% sodium hypochlorite solution from the Erlenmeyer flask into the sieve until it is approximately half full (Figure D-3). Be careful not to overfill the sieve as that will cause the eggs to spill out over the top of the sieve. Set the timer for 1 minute and press start. After 1 minute, gently remove the sieve from the nesting soaking pan. Rinse the eggs by running tap water over them for 1 minute (set timer as before). Tilt the sieve containing the eggs at a 45 degree angle and gently wash the eggs to the bottom portion of the sieve (Figure D-4). Once the eggs are gathered at the bottom of the sieve, hold it at a 110 degree angle over a clean ovipot pot and squirt distilled water to dislodge the eggs from the sieve, distributing them as evenly as possible back into one or more clean, dry ovipots (Figure D-5). Place clean vented lids on the ovipots and label each with the set-up date and 1% sodium hypochlorite to indicate that the eggs have been washed or other indication that the eggs have been washed with sodium hypochlorite. Repeat this process with each ovipot, then set the pots on lab towels to soak up excess water. Put pots into white plastic trays according to species and store them on shelves in an incubator at 25°C and 70-80% RH.
